# Long-term efficacy and reintervention of focused ultrasound ablation surgery monotherapy for adenomyosis: a systematic review and meta-analysis

**DOI:** 10.3389/fmed.2026.1855726

**Published:** 2026-06-12

**Authors:** Liang Hu, Danfeng Shi, Meijie Yang, Zhaorui Zou, Jinyun Chen

**Affiliations:** 1 State Key Laboratory of Ultrasound in Medicine and Engineering, College of Biomedical Engineering, Chongqing Medical University, Chongqing, China; 2 Department of Gynaecology, Chongqing Haifu Hospital, Chongqing, China; 3 Department of Radiology, Chongqing Hospital of Traditional Chinese Medicine, Chongqing, China; 4 College of Artificial Intelligence Medicine, Chongqing Medical University, Chongqing, China

**Keywords:** adenomyosis, focused ultrasound ablation surgery, long-term efficacy, pregnancy, reintervention

## Abstract

**Background:**

The clinical management of adenomyosis remains challenging. Pharmacological therapies and surgical approaches for adenomyosis have shown benefits but present important limitations, such as side effects, recurrence, and loss of fertility. In recent years, focused ultrasound ablation surgery (FUAS) has emerged as a promising uterus-preserving alternative. Nevertheless, it has not yet been approved by major regulatory authorities worldwide for the treatment of adenomyosis, and relevant evidence on the long-term efficacy of FUAS as well as the need for reintervention is scarce.

**Objective:**

Aimed to systematically evaluate the long-term outcomes and reinterventions of FUAS for adenomyosis.

**Methods:**

A comprehensive literature search was conducted in PubMed, Embase, Web of Science, and the Cochrane Library to identify relevant studies published up to February 2026, which investigated the clinical outcomes of adenomyosis patients undergoing FUAS monotherapy. Study selection and data extraction were independently performed by two researchers each. Fixed-effect and random-effect models were utilized to calculate synthesized effect sizes. Mean differences (MDs) were used for continuous outcomes, and proportions were used to assess dichotomous outcomes.

**Results:**

A total of 27 studies involving 13,307 patients were included in this review. The pooled nonperfused volume ratio (NPVR) was 70%. The reduction in dysmenorrhea VAS score was 3.52 (95% CI: 3.15–3.90), with a dysmenorrhea remission rate of 0.79 (95% CI: 0.75–0.83). The reduction in menorrhagia severity score was 1.31 (95% CI: 1.21–1.40), with a menorrhagia remission rate of 0.75 (95% CI: 0.69–0.81). The female sexual function index (FSFI) score increased by 5.38 (95% CI: 2.77–8.00). All improvements were statistically significant (*p <* 0.001). The incidence of major adverse events was 0.3%, whereas minor adverse events occurred in 46% of cases. The pooled pregnancy rate was 6%. The overall reintervention and recurrence rates were 7 and 20%, respectively, while the long-term reintervention and recurrence rates were 8 and 45%, respectively.

**Conclusion:**

FUAS is a noninvasive, safe, and effective treatment for adenomyosis, demonstrating sustained long-term efficacy with a favorable reintervention rate.

**Systematic review registration:**

https://www.crd.york.ac.uk/PROSPERO/view/CRD420261329888.

## 1 Introduction

Adenomyosis represents a gynecological disorder featured by the ectopic invasion of endometrial glands and stroma into the myometrium (1). This benign uterine condition is frequently observed in women of reproductive age, with a prevalence rate ranging from 10 to 17% (2, 3). It manifests through symptoms such as painful menstruation, excessive menstrual bleeding, persistent pelvic pain, and reduced fertility, all of which greatly diminish the patients’ quality of life (4).

Treatment options for adenomyosis typically involve surgery, minimally invasive procedures and medication. Surgical procedures, such as hysterectomy and laparoscopic excision (LE), come with numerous limitations and side effects. These procedures can be quite invasive, with potential complications like infection, bleeding, and the formation of adhesions. It also affects the structure and function of the uterus and may lead to loss of fertility (5, 6). Minimally invasive treatments for adenomyosis, including uterine artery embolization, microwave ablation, and radiofrequency ablation, remain controversial for potential effects on ovarian function, and are still limited by a lack of long-term clinical data (7–9). Medical therapies include gonadotropin-releasing hormone agonists (GnRHa), contraceptives, aromatase inhibitors and others, however, they cannot be used long-term because of risks such as increased cardiovascular events and osteoporosis (10).

FUAS also known as high-intensity focused ultrasound (HIFU), is a noninvasive treatment method that preserves organs and is mainly utilized for addressing solid tumors (11–13). In recent years, it has been increasingly applied in the management of adenomyosis (14–16). However, Existing studies mainly focus on short-term follow-up findings, and the evidence related to long-term efficacy and reintervention rates of FUAS serving as monotherapy for adenomyosis is still insufficient and inconsistent. In addition, despite previous effective and safe data, FUAS has not yet been approved for the indication of adenomyosis by major regulatory authorities worldwide such as the FDA, National Medical Products Administration (NMPA), and European authorities. Therefore, high-quality and high-level evidence is both scientifically rigorous and clinically necessary. This will improve the precision, reliability, and durability of estimating long-term outcomes and fill the systematic evidence gap, which is urgently needed for clinical decision-making, guideline development, and approval by regulatory authorities. Therefore, this study carried out a systematic review and meta-analysis to investigate the long-term efficacy and reintervention of FUAS for adenomyosis.

## 2 Materials and methods

### 2.1 Study design

The protocol for this systematic review and meta-analysis was registered in the PROSPERO database (registration number: CRD420261329888) and conducted in strict accordance with the reporting guidelines of the Preferred Reporting Items for Systematic Reviews and Meta-Analyses (PRISMA) statements (17) (Supplementary Table S2). This research aimed to examine the long-term effects of FUAS on adenomyosis. In studies involving different interventions, only the data from the FUAS group were considered for analysis. PICOS Framework was used in the present study design: Population (P): Adult women with symptomatic adenomyosis. Intervention (I): FUAS monotherapy (i.e., FUAS without concomitant surgical or pharmacological treatment for adenomyosis). Comparator (C): No comparator group required (single-arm meta-analysis); for studies with a comparator arm, any alternative treatment or no treatment. Outcomes (O): Primary: dysmenorrhea VAS score, dysmenorrhea remission rate, menorrhagia severity score, menorrhagia remission rate, adverse events, recurrence rate, and reintervention rate; secondary: symptom severity score (SSS), quality of life (QoL), FSFI score, NPVR, reduction in volume of lesion and uterus, CA125 levels, and pregnancy rate. Study design (S): Randomized controlled trials, non-randomized controlled trials, prospective or retrospective cohort studies, case–control studies, and case series.

### 2.2 Study outcome measures

Dysmenorrhea was assessed using the visual analog scale (VAS) to evaluate the severity or improvement of pain before and after treatment, where 0 indicates no pain and 10 indicates unbearable pain.

The remission rate of dysmenorrhea was defined as the proportion of patients whose dysmenorrhea symptoms were alleviated or resolved after FUAS treatment compared with the baseline, and it was used to objectively reflect the effectiveness of the procedure in improving pain symptoms.

The menorrhagia severity score is a quantitative indicator used in clinical studies of adenomyosis to evaluate the severity of heavy menstrual bleeding and its improvement after treatment. In the studies that were part of this systematic review, a 5-point scale was utilized, where a score of 1 represented no menorrhagia and a score of 5 represented severe menorrhagia.

The remission rate of menorrhagia was defined as the percentage of patients who experienced an improvement in menstrual blood loss compared to the baseline after undergoing treatment.

Adverse events were classified according to the Society of Interventional Radiology (SIR) grading system (grades A–F). Grades A-B were defined as minor adverse events that were resolved spontaneously without treatment or required minimal therapy. Grades C-F were defined as major adverse events, including those requiring hospitalization for less than 48 h, significant therapeutic intervention, permanent sequelae, or death (18).

The recurrence rate was defined as the proportion of patients who experienced a return of symptoms or had imaging evidence of adenomyotic lesion regrowth after treatment.

The reintervention rate was characterized as the percentage of patients needing further treatment due to recurrence, which included undergoing another FUAS, uterine artery embolization (UAE), lesion removal, hysterectomy, or receiving a levonorgestrel-releasing intrauterine device or a gonadotropin-releasing hormone agonist (GnRHa).

The SSS was employed to assess symptoms associated with adenomyosis, including dysmenorrhea, irregular menstrual cycles, and pelvic discomfort. The scoring ranges from 0 to 100, where higher scores denote more intense symptoms.

The Quality of Life (UFS-QoL) questionnaire is a specialized tool designed for assessing benign uterine conditions (19). It uses a range of scoring from 0 to 100, where higher scores reflect an improved quality of life. The FSFI score was employed to evaluate sexual function, with greater total scores signifying enhanced sexual functioning. To measure improvements in sexual function, changes in FSFI scores were compared before and after treatment.

Changes in uterine and adenomyosis lesion volumes before and after treatment were evaluated using ultrasound or MRI: Uterine volume reduction = uterine volume before treatment—uterine volume after treatment; lesion volume reduction = lesion volume before treatment—lesion volume after treatment.

Uterine and lesion volumes are typically calculated by measuring three orthogonal diameters (0.5233 × length × width × thickness) on MRI or ultrasound using the ellipsoid volume formula.

NPVR is defined as the percentage of non-perfused (necrotic) volume within the adenomyotic lesion, as shown on contrast-enhanced MRI or ultrasound after FUAS treatment. It is used to quantitatively assess the extent of lesion ablation and is a key indicator of the local therapeutic effect of the FUAS. NPVR (%) = (non-perfused volume/total lesion volume) × 100%.

Serum CA125 reflects the activity of adenomyosis and the therapeutic response to treatment.

Pregnancy rate: Defined as the proportion of patients who achieved pregnancy after FUAS treatment. Pregnancy rate = (number of pregnancies/total number of treated patients) × 100%.

### 2.3 Inclusion and exclusion criteria

Inclusion criteria: (1) The study population consisted of patients with a confirmed diagnosis of adenomyosis without coexisting uterine fibroids, uterine sarcoma, or other benign or malignant uterine diseases and without contraindications to FUAS treatment, such as severe hepatic or renal dysfunction or coagulation disorders. The objective of this study was to evaluate the safety, effectiveness, and reintervention of FUAS in the treatment of adenomyosis; (2) Patients received FUAS as monotherapy without concomitant pharmacological or surgical treatments. For controlled studies, only data from the FUAS group were included; (3) Randomized controlled trials (RCTs), non-randomized controlled clinical trials (non-RCTs), cohort studies (prospective or retrospective), case–control and case series studies with a sample size of ≥20 were included. Only studies published in English were considered; (4). Studies reporting at least one of the predefined primary outcomes and providing sufficient data (e.g., sample size, number of events, means, and standard deviations) were included.

Exclusion criteria: (1) Case reports, review articles, animal studies, basic research, trial protocols, secondary analyses, letters, replies, and conference summaries were excluded; (2) Duplicate publications and studies without primary outcome data or from which primary outcome data could not be extracted were also excluded.

### 2.4 Literature retrieval and data extraction

The search strategy of this study combined key words and free words. The literature retrieval was performed across PubMed, Embase, Cochrane Library, and Web of Science. The search period covered database inception to February 2026. In addition, to minimize the risk of missing relevant studies, the review articles and meta-analyses were cross-referenced. The PubMed search string was constructed as follows: (adenomyosis OR adenomyoma) AND ((high-intensity focused ultrasound ablation[MeSH Terms]) OR (focused ultrasound[Title/Abstract]) OR (HIFU[Title/Abstract]) OR (magnetic resonance-guided focused ultrasound surgery[Title/Abstract]) OR (MRgFUS[Title/Abstract]) OR (FUAS[Title/Abstract]) OR (MRgHIFU[Title/Abstract]) OR (USgHIFU[Title/Abstract])), and the complete search strategy was summarized in Supplementary Table S1. Two researchers independently examined the titles and abstracts of all the studies retrieved to exclude those that did not satisfy the inclusion criteria. The full texts of studies that appeared to be eligible were then acquired and thoroughly evaluated to finalize their inclusion. A PRISMA flow diagram was created to depict the study selection process. A standardized data extraction form was designed to gather information on the primary and secondary outcomes. Any discrepancies between the two researchers were settled through discussion with a third researcher. The selection process and the reasons for exclusion were also recorded.

### 2.5 Quality and risk of bias assessment

The Methodological Index for Non-Randomized Studies (MINORS) was utilized to determine the quality of single-arm studies and non-RCTs (20). This tool includes 12 items, with a scoring system where 0 points are given if not reported, 1 point if reported but insufficient, and 2 points if reported and sufficient, culminating in a maximum score of 24. The initial eight items pertain to single-arm studies, whereas all 12 items are relevant for non-RCTs.

### 2.6 Statistical analysis

Stata 16.0 (Stata Corp LLC, College Station, TX, USA) was adopted for all statistical analyses. To evaluate heterogeneity among the studies included Cochran’s *Q* test and the *I*^2^ statistic were utilized. Heterogeneity was deemed low if *p* was greater than 0.1 and *I*^2^ was 50% or less, prompting the use of a fixed-effects model for the meta-analysis. Conversely, if *p* was 0.1 or less or *I*^2^ exceeded 50%, heterogeneity was considered significant, and a random-effects model was utilized. For continuous variables such as dysmenorrhea score, menorrhagia score, SSS, and QoL, the mean difference (MD) with 95% confidence intervals (CI) was used. For dichotomous variables like response rate, recurrence rate, reintervention rate, and incidence of adverse events, proportions (rates) with 95% CI served as pooled effect sizes. Long-term follow-up was defined as ≥24 months post-FUAS, in line with the 24-month/2-year follow-up endpoints commonly used to assess sustained outcomes after gynecologic minimally invasive therapies (21, 22). Subgroup analyses based on follow-up time points were performed to examine the consistency of results over time and to identify variations between subgroups.

To evaluate the stability of the pooled outcomes, sensitivity analysis was conducted by omitting individual studies one by one and recalculating the overall effect size. If low-quality studies were identified, the analyses were repeated after their exclusion to evaluate their impact on the pooled estimates. To conclude, funnel plots were utilized to examine reporting bias, and Begg’s or Egger’s tests were conducted to detect any potential publication bias when the study count reached 10 or more. In cases where significant publication bias was found, the trim-and-fill method was applied to determine its influence on the outcomes. All statistical tests were two-tailed, and a *p*-value < 0.05 was considered indicative of statistical significance.

## 3 Results

### 3.1 Study selection

**Figure 1:**
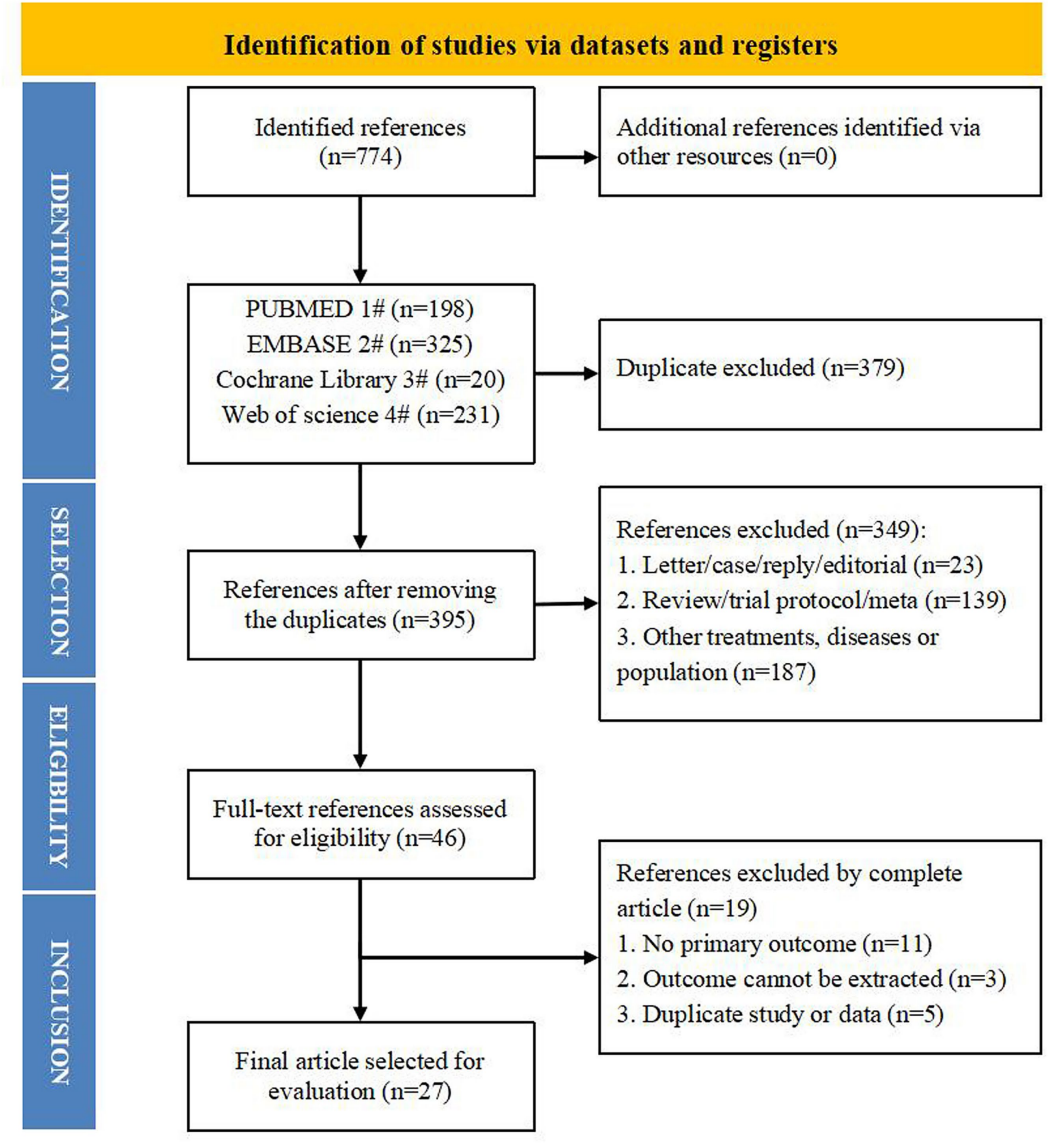
The PRISMA flowchart of study retrieval and selection.

A total of 774 records were identified through searches of the four predefined databases, of which 379 duplicates were removed. In line with established eligibility criteria, 343 studies were excluded during title and abstract screening. The remaining articles were assessed through full-text review, and 19 studies were excluded because of the absence of primary outcomes, inability to extract data, or duplication of studies or datasets. Ultimately, 27 studies involving 13,307 patients were included in this meta-analysis (Table 1). The literature search and selection processes are illustrated in Figure 1.

**Table 1:** Baseline characteristics of the studies included in this review.

No.	Author	Year	Study type	Sample	Follow-up	Outcomes	Age (years)	BMI
1	Zhao (67)	2026	non-RCT	75	12mo	①, ⑦, ⑩	45.5 ± 3.8	23.5 ± 3.4
2	Lin (68)	2025	non-RCT	396	58mo	②, ⑫, ⑬, ⑭	40.0 (36.0, 44.0)	NA
3	Jin (69)	2025	non-RCT	35	12mo	①, ⑤, ⑥, ⑧, ⑫, ⑬	45.5 ± 4.8	23.2 ± 3.5
4	Wu (70)	2024	non-RCT	69	12mo	①, ②, ③	39.7 ± 5.5	NA
5	Fan (53)	2024	SAT	238	15mo	②, ④, ⑭	40.8 ± 5.9	NA
6	Zhu (71)	2023	non-RCT	34	24mo	①, ③, ⑧, ⑨, ⑪, ⑫	42.94 ± 5.5	NA
7	Zhao (35)	2023	non-RCT	227	6mo	①, ②, ③, ④, ⑧, ⑩, ⑫	43.0 (19.0, 52.0)	NA
8	Xu (37)	2023	non-RCT	405	18mo	①, ②, ③, ④, ⑧, ⑨, ⑩, ⑫	41.1 ± 6.2	NA
9	Xu (72)	2021	SAT	84	12mo	①, ②, ⑧, ⑫	41.4 ± 5.5	NA
10	Li (73)	2021	non-RCT	485	60mo	①, ②, ③, ④, ⑧, ⑫, ⑬, ⑮	40.6 ± 5.5	22.6 ± 3.0
11	Li (74)	2020	SAT	67	12mo	①, ③, ⑩, ⑮	38. ± 5.3	23.0 ± 3.1
12	Jeng (75)	2020	SAT	149	3mo	①, ⑤, ⑦, ⑨, ⑩, ⑪, ⑫, ⑮	41.7 ± 5.3	23.2 ± 4.5
13	Huang (23)	2020	non-RCT	50	12mo	①, ③, ⑨, ⑮	36.0 (32.0, 38.0)	21.4(19.7,23.5)
14	Lee (26)	2019	SAT	889	12mo	⑤, ⑥, ⑨, ⑫, ⑬, ⑭, ⑮	41.1 ± 5.5	NA
15	Zhang (76)	2018	non-RCT	38	12mo	①, ②, ⑧, ⑫	41.2 ± 7.1	23.9 ± 1.1
16	Liu (41)	2018	SAT	8,434	NA	⑫	NA	NA
17	Keserci (38)	2018	non-RCT	66	6mo	⑤, ⑧, ⑩, ⑫	NA	NA
18	Guo (24)	2018	non-RCT	45	12mo	①, ③, ⑧, ⑨, ⑩, ⑫, ⑬, ⑭, ⑮	42.4 ± 5.1	NA
19	Feng(a) (77)	2017	non-RCT	417	NA	⑧, ⑫	38.9 ± 5.7	22.4 ± 2.6
20	Feng(b) (78)	2017	non-RCT	297	36mo	①, ②, ③, ④, ⑧	NA	NA
21	Liu (79)	2016	SAT	208	40mo	①, ②, ⑧, ⑫, ⑭	39.6 ± 5.1	22.0 ± 2.4
22	Shui (22)	2015	SAT	224	24mo	①, ②, ③, ④, ⑧, ⑫	41.6 ± 4.6	NA
23	Long (80)	2015	SAT	47	12mo	①, ⑤, ⑦, ⑧, ⑨, ⑩, ⑫, ⑮	37.4 ± 5.1	22.0 ± 1.7
24	Zhang (81)	2014	non-RCT	202	18mo	①, ②, ③, ④, ⑧, ⑫	41.7 ± 4.9	NA
25	Zhou (82)	2011	SAT	78	24mo	①, ③, ⑧, ⑫, ⑭	37.7 ± 4.9	NA
26	Kim (25)	2011	SAT	28	6mo	①, ⑤, ⑫, ⑮	42.0 ± 6.0	22.2 ± 2.1
27	Fukunishi (83)	2008	SAT	20	6mo	⑤, ⑨, ⑩, ⑫, ⑮	42.5 ± 3.9	21.8 ± 3.6

SAT, single arm trial; non-RCT, non-randomised controlled trial; NA, not applicable; mo, months; outcome measures, ① dysmenorrhea VAS score; ② dysmenorrhea remission rate; ③ menorrhagia severity score; ④ menorrhagia remission rate; ⑤ symptom severity score (SSS); ⑥ quality of life (QoL) score; ⑦ female sexual function index (FSFI) score; ⑧ nonperfused volume ratio (NPVR); ⑨ reduction volume of uterus; ⑩ reduction volume of lesion; ⑪ CA125; ⑫ adverse reaction; ⑬ reintervention rate; ⑭ recurrence rate; ⑮ pregnancy rate.

### 3.2 Study quality assessment

Among the 27 included studies, 12 were single-arm studies with MINORS scores ranging from 11 to 14, indicating moderate to high quality, The main causes for the decrease in score were the absence of prospective data gathering and the failure to determine the sample size. The remaining 15 studies were non-randomized controlled trials, with MINORS scores ranging from 19 to 24, also reflecting moderate to high quality, the absence of prospective data gathering and the failure to determine the sample size (Table 2).

**Table 2:** The MINORS scores of the included studies.

No.	Author	Year	I	II	III	IV	V	VI	VII	VII	IX	X	XI	XII	Total
1	Zhao (67)	2026	2	2	0	2	2	2	2	2	2	2	2	2	22
2	Lin (68)	2025	2	2	0	2	2	2	2	0	2	2	2	2	20
3	Jin (69)	2025	2	2	0	2	2	2	2	2	2	2	2	2	22
4	Wu (70)	2024	2	2	0	2	2	2	2	0	2	2	1	2	19
5	Fan (53)	2024	2	2	0	2	2	2	1	0	NA	NA	NA	NA	11
6	Zhu (71)	2023	2	2	0	2	2	2	2	0	2	2	2	2	20
7	Zhao (35)	2023	2	2	0	2	2	2	2	0	2	2	2	2	20
8	Xu (37)	2023	2	2	0	2	2	2	2	0	2	2	1	2	19
9	Xu (72)	2021	2	2	0	2	2	2	2	0	NA	NA	NA	NA	12
10	Li (73)	2021	2	2	0	2	2	2	1	0	2	2	0	2	17
11	Li (74)	2020	2	2	0	2	2	2	1	0	NA	NA	NA	NA	11
12	Jeng (75)	2020	2	2	0	2	2	2	2	0	NA	NA	NA	NA	12
13	Huang (23)	2020	2	2	0	2	2	2	2	0	2	2	2	2	20
14	Lee (26)	2019	2	2	0	2	2	2	2	0	NA	NA	NA	NA	12
15	Zhang (76)	2018	2	2	2	2	2	2	1	0	2	2	2	2	21
16	Liu (41)	2019	2	2	0	2	2	2	2	0	NA	NA	NA	NA	12
17	Keserci (38)	2018	2	2	0	2	2	2	2	2	2	2	2	2	22
18	Guo (24)	2018	2	2	0	2	2	2	2	0	2	2	2	2	20
19	Feng(a) (77)	2017	2	2	0	2	2	2	2	0	2	2	2	2	20
20	Feng(b) (78)	2017	2	2	0	2	2	2	2	0	2	2	2	2	20
21	Liu (79)	2016	2	2	0	2	2	2	1	0	NA	NA	NA	NA	11
22	Shui (22)	2015	2	2	0	2	2	2	2	0	NA	NA	NA	NA	12
23	Long (80)	2015	2	2	0	2	2	2	2	0	NA	NA	NA	NA	12
24	Zhang (81)	2014	2	2	0	2	2	2	1	0	2	2	2	2	19
25	Zhou (82)	2011	2	2	2	2	2	2	1	0	NA	NA	NA	NA	13
26	Kim (25)	2011	2	2	0	2	2	2	1	0	NA	NA	NA	NA	11
27	Fukunishi (83)	2008	2	2	2	2	2	2	2	0	NA	NA	NA	NA	14

No. 5, 10, 11, 13, 14, 15, 21, 22, 23, 25, 26, and 27 studies were single-arm studies, while the remaining studies were non-randomized controlled trials; NA, not applicable; Items I-XII indicate: I, A clearly stated aim; II, Inclusion of consecutive patients; III, Prospective collection of data; IV, Endpoints appropriate to reflect the study aim; V, Objective assessment of endpoints; VI, Adequacy of follow-up; VII, Loss to follow-up rate <5%; VII, A prospective sample size calculation; IX, An adequate control group; X, Contemporary group; XI, Baseline equivalence of groups; and XII, Adequate statistical analysis.

### 3.3 Symptoms, quality of life, and sexual function

#### 3.3.1 Dysmenorrhea VAS score

**Figure 2:**
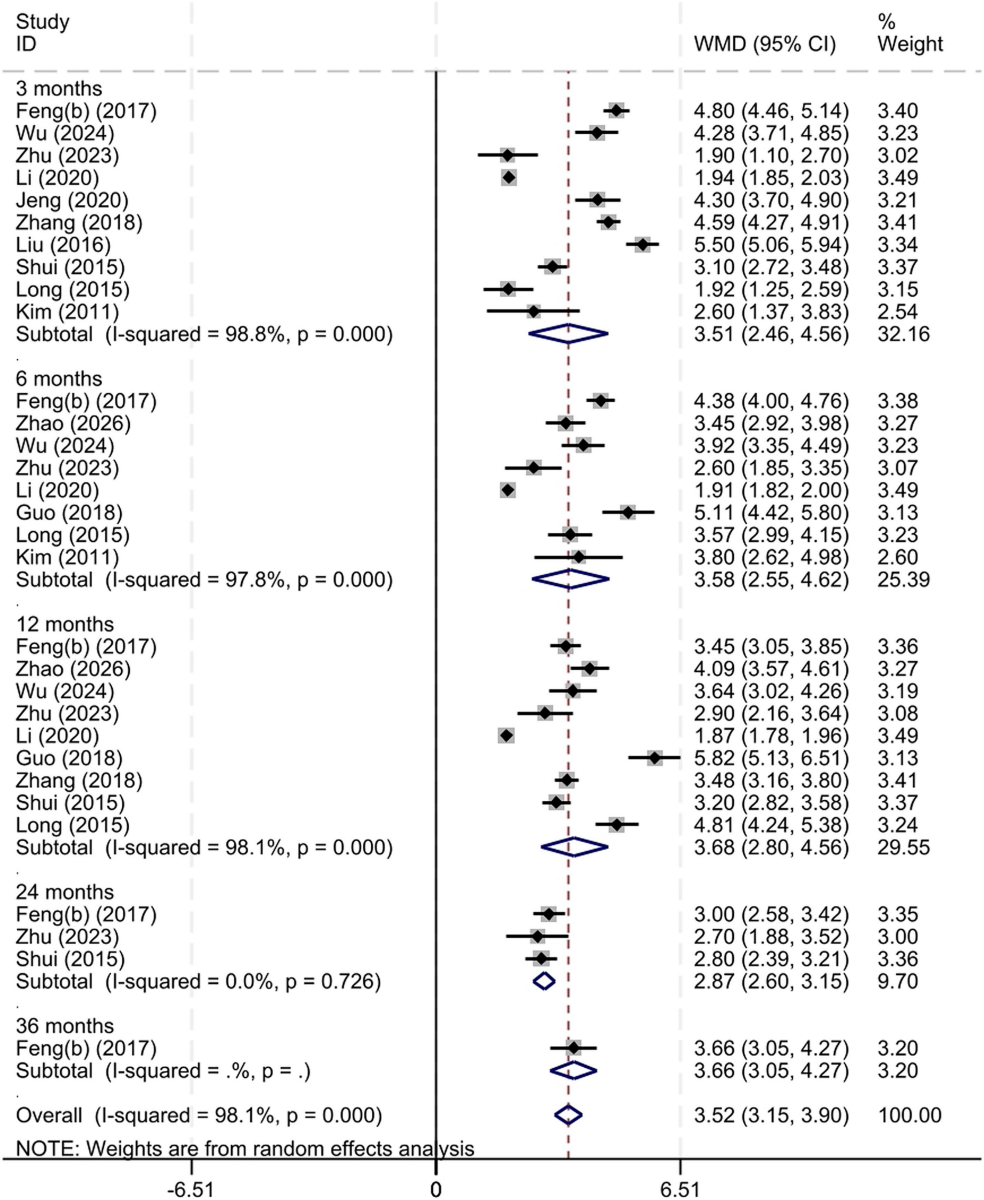
Forest plot of dysmenorrhea VAS score.

Twelve studies reported dysmenorrhea VAS scores. Across all follow-up time points (3–36 months), FUAS significantly improved dysmenorrhea compared with the baseline. Substantial heterogeneity was identified (*I*^2^ = 98.1%, *p <* 0.001); therefore, a random-effects model was used. The pooled reduction in VAS score of WMD was 3.52 (95% CI: 3.15, 3.90). The reductions at 3, 6, 12, 24, and 36 months were 3.51 (95% CI: 2.46, 4.56), 3.58 (95% CI: 2.55, 4.62), 3.68 (95% CI: 2.80, 4.56), 2.87 (95% CI: 2.60, 3.15), and 3.66 (95% CI: 3.05, 4.27), respectively (all *p <* 0.001) (Figure 2).

#### 3.3.2 Dysmenorrhea remission rate

**Figure 3:**
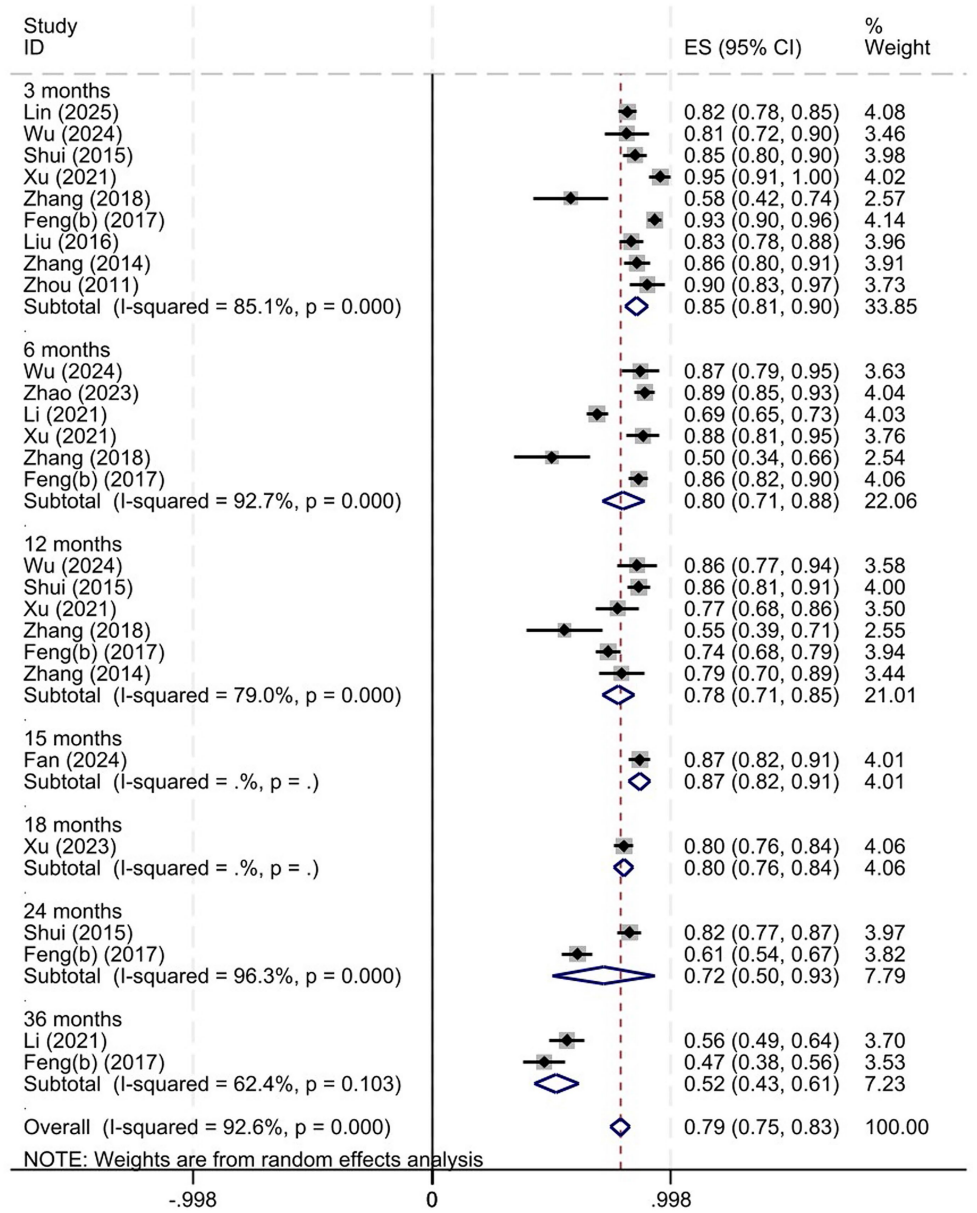
Forest plot of dysmenorrhea remission rate.

Fourteen studies reported dysmenorrhea remission rates. At all follow-up time points (3–36 months), the remission rate after FUAS improved significantly compared with the baseline. Due to substantial heterogeneity (*I*^2^ = 92.6%, *p <* 0.001), a random-effects model was used. The pooled remission rate was 0.79 (95% CI: 0.75, 0.83; *p <* 0.001). The remission rates at 3, 6, 12, 15, 18, 24, and 36 months were 0.85 (95% CI: 0.81, 0.90), 0.80 (95% CI: 0.71, 0.88), 0.78 (95% CI: 0.71, 0.85), 0.87 (95% CI: 0.82, 0.91), 0.80 (95% CI: 0.76, 0.84), 0.72 (95% CI: 0.50, 0.93), and 0.52 (95% CI: 0.43, 0.61), respectively (all *p <* 0.001) (Figure 3).

#### 3.3.3 Menorrhagia severity score

**Figure 4:**
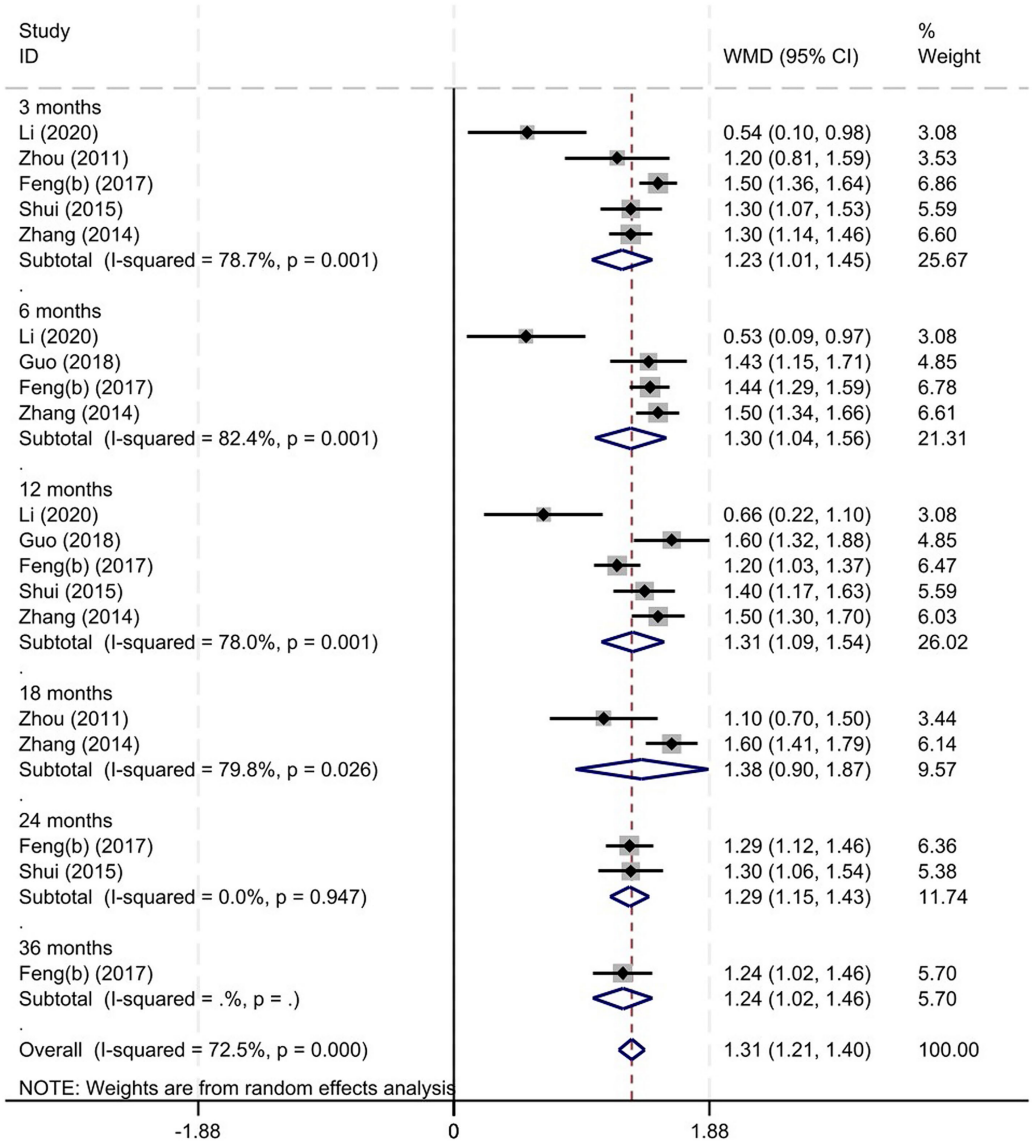
Forest plot of menorrhagia severity score.

Six studies reported menorrhagia severity scores. Significant improvement compared with the baseline was observed at all follow-up time points (3–36 months). Moderate heterogeneity was present (*I*^2^ = 72.5%, *p <* 0.001), and a random-effects model was used. The pooled reduction in menorrhagia severity score of WMD was 1.31 (95% CI: 1.21, 1.40; *p <* 0.001). Reductions at 3, 6, 12, 18, 24, and 36 months were 1.23 (95% CI: 1.01, 1.45; *p <* 0.001), 1.30 (95% CI: 1.04, 1.56; *p <* 0.001), 1.31 (95% CI: 1.09, 1.54; *p <* 0.001), 1.38 (95% CI: 0.90, 1.87; *p <* 0.001), 1.29 (95% CI: 1.15, 1.43; *p <* 0.001), and 1.24 (95% CI: 1.02, 1.46; *p =* 0.030), respectively (Figure 4).

#### 3.3.4 Menorrhagia remission rate

**Figure 5:**
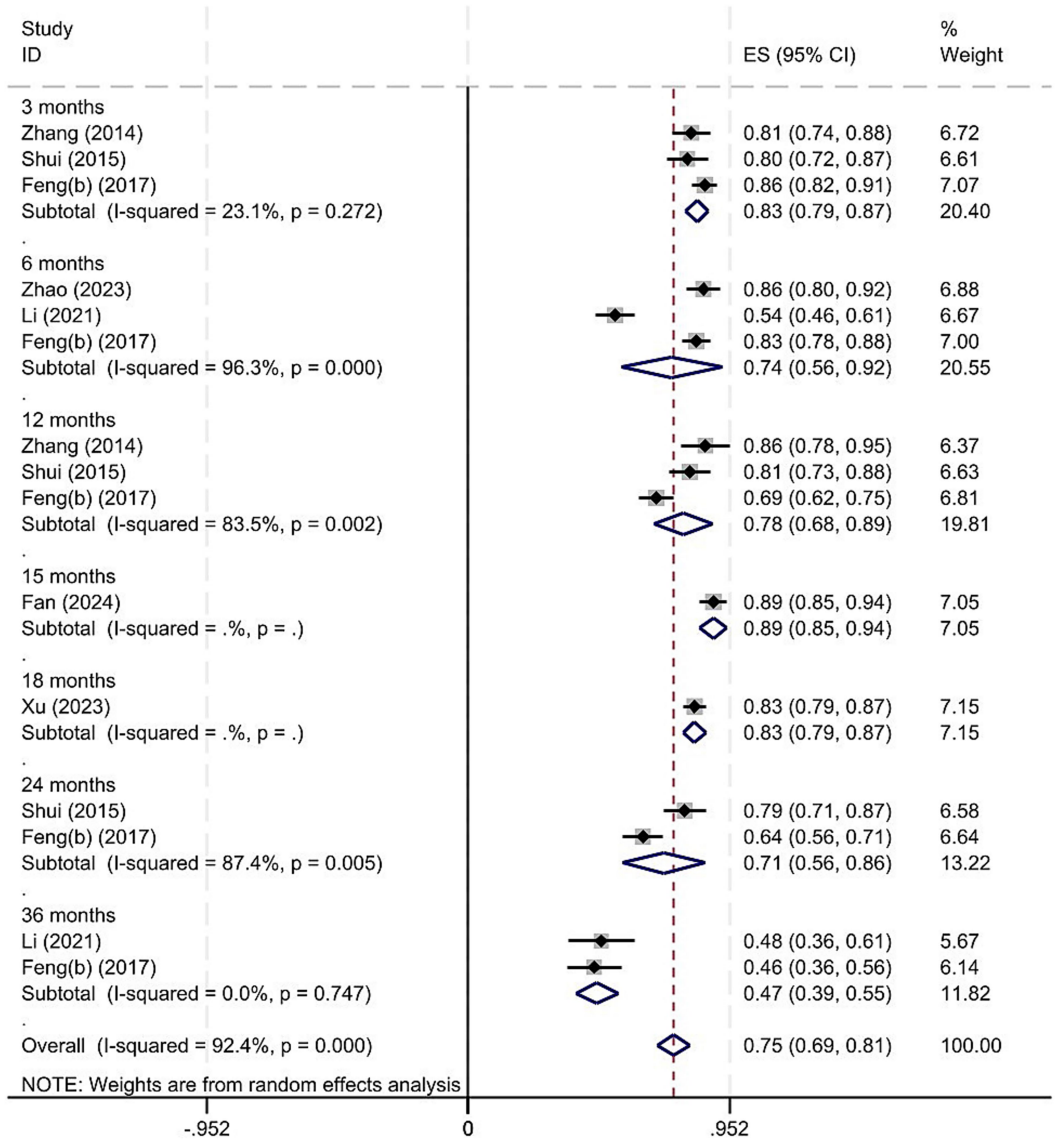
Forest plot of menorrhagia remission rate.

Eight studies reported the improvement rate of menorrhagia. Significant remission rates were observed at all follow-up time points (3–36 months). Given the substantial heterogeneity (*I*^2^ = 92.4%, *p <* 0.001), a random-effects model was used. The pooled remission rate was 0.75 (95% CI: 0.69, 0.81; *p <* 0.001). Remission rates at 3, 6, 12, 15, 18, 24, and 36 months were 0.83 (95% CI: 0.79, 0.87), 0.74 (95% CI: 0.56, 0.92), 0.78 (95% CI: 0.68, 0.89), 0.89 (95% CI: 0.85, 0.94), 0.83 (95% CI: 0.79, 0.87), 0.71 (95% CI: 0.56, 0.86), and 0.47 (95% CI: 0.39, 0.55), respectively (all *p <* 0.001) (Figure 5).

#### 3.3.5 Symptom severity score (SSS)

Six studies reported the SSS outcomes. Significant reductions were observed at all follow-up time points (3–12 months). With substantial heterogeneity (*I*^2^ = 83.4%, *p <* 0.001), a random-effects model was employed. The pooled reduction in SSS of WMD was 25.61 (95% CI: 22.86, 28.37; *p <* 0.001). Reductions at 3, 6, and 12 months were 20.48 (95% CI: 13.95, 27.01), 28.23 (95% CI: 21.97, 34.49), and 26.68 (95% CI: 20.63, 32.74), respectively (all *p <* 0.001) (Supplementary Figure S1).

#### 3.3.6 Quality of life (QoL)

Two studies reported QoL outcomes. Significant improvements were observed at all follow-up time points (3–12 months). Given the minimal heterogeneity (*I*^2^ = 2.3%, *p =* 0.394), a fixed-effects model was used. The pooled improvement in QoL of WMD was 21.39 (95% CI: 20.34, 22.44; *p <* 0.001). Improvements at 3, 6, and 12 months were 20.36 (95% CI: 18.44, 22.28), 21.99 (95% CI: 20.18, 23.81), and 21.69 (95% CI: 19.94, 23.34), respectively (all *p <* 0.001) (Supplementary Figure S2).

#### 3.3.7 Sexual function (FSFI)

Three studies reported FSFI outcomes. Significant improvements were observed at 6 and 12 months. Due to substantial heterogeneity (*I*^2^ = 96.2%, *p <* 0.001), a random-effects model was applied. The pooled improvement in FSFI of WMD was 5.38 (95% CI: 2.77, 8.00; *p <* 0.001). Changes at 3, 6, and 12 months were −0.25 (95% CI: −8.55, 8.04; *p =* 0.952), 5.82 (95% CI: 3.87, 7.77; *p <* 0.001), and 8.47 (95% CI: 6.04, 10.90; *p <* 0.001), respectively (Supplementary Figure S3).

### 3.4 Imaging and biochemical outcomes

#### 3.4.1 Non-perfused volume ratio (NPVR)

Thirteen studies reported NPVR. Moderate heterogeneity was observed (*I*^2^ = 66.5%, *p <* 0.001), and a random-effects model showed a pooled NPVR of 0.70 (95% CI: 0.66, 0.73; *p <* 0.001) (Supplementary Figure S4).

#### 3.4.2 Reduction volume of uterus

Five studies reported uterine volume reduction. Significant reductions were observed at 3 months. With substantial heterogeneity (*I*^2^ = 98.6%, *p <* 0.001), a random-effects model yielded a pooled reduction of WMD = 68.02 (95% CI: 38.79, 97.25; *p <* 0.001). Reductions at 3, 6, 12, and 24 months were 90.09 (95% CI: 24.24, 155.95; *p =* 0.007), 63.64 (95% CI: −5.09, 132.38; *p =* 0.070), 72.89 (95% CI: −7.99, 153.77; *p =* 0.077), and 3.90 (95% CI: −33.57, 41.37; *p =* 0.838), respectively.

#### 3.4.3 Reduction volume of lesion

Five studies reported lesion volume reduction. Significant reductions were observed at 6–12 months. Moderate heterogeneity was present (*I*^2^ = 48.8%, *p =* 0.068), and the pooled reduction in lesion volume of WMD was 27.54 (95% CI: 19.90, 35.18; *p <* 0.001). Reductions at 3, 6, and 12 months were 75.20 (95% CI: −7.82, 158.22; *p =* 0.076), 22.66 (95% CI: 17.22, 28.10; *p <* 0.001), and 34.45 (95% CI: 28.93, 39.98; *p <* 0.001), respectively.

#### 3.4.4 Serum CA125

Two studies reported CA125 levels. Significant reductions were observed at 6–12 months. Moderate heterogeneity was present but was not statistically significant (*I*^2^ = 46.3%, *p =* 0.114). The pooled reduction in CA125 level of WMD was 26.40 (95% CI: 13.53, 39.28; *p <* 0.001). Reductions at 3, 6, 12, and 24 months were 49.16 (95% CI: −8.85, 107.17; *p =* 0.097), 27.20 (95% CI: 9.29, 45.11; *p =* 0.003), 25.83 (95% CI: 7.88, 43.78; *p =* 0.005), and 12.92 (95% CI: −6.56, 32.40; *p =* 0.194), respectively.

### 3.5 Safety outcomes

#### 3.5.1 Minor adverse events

**Figure 6:**
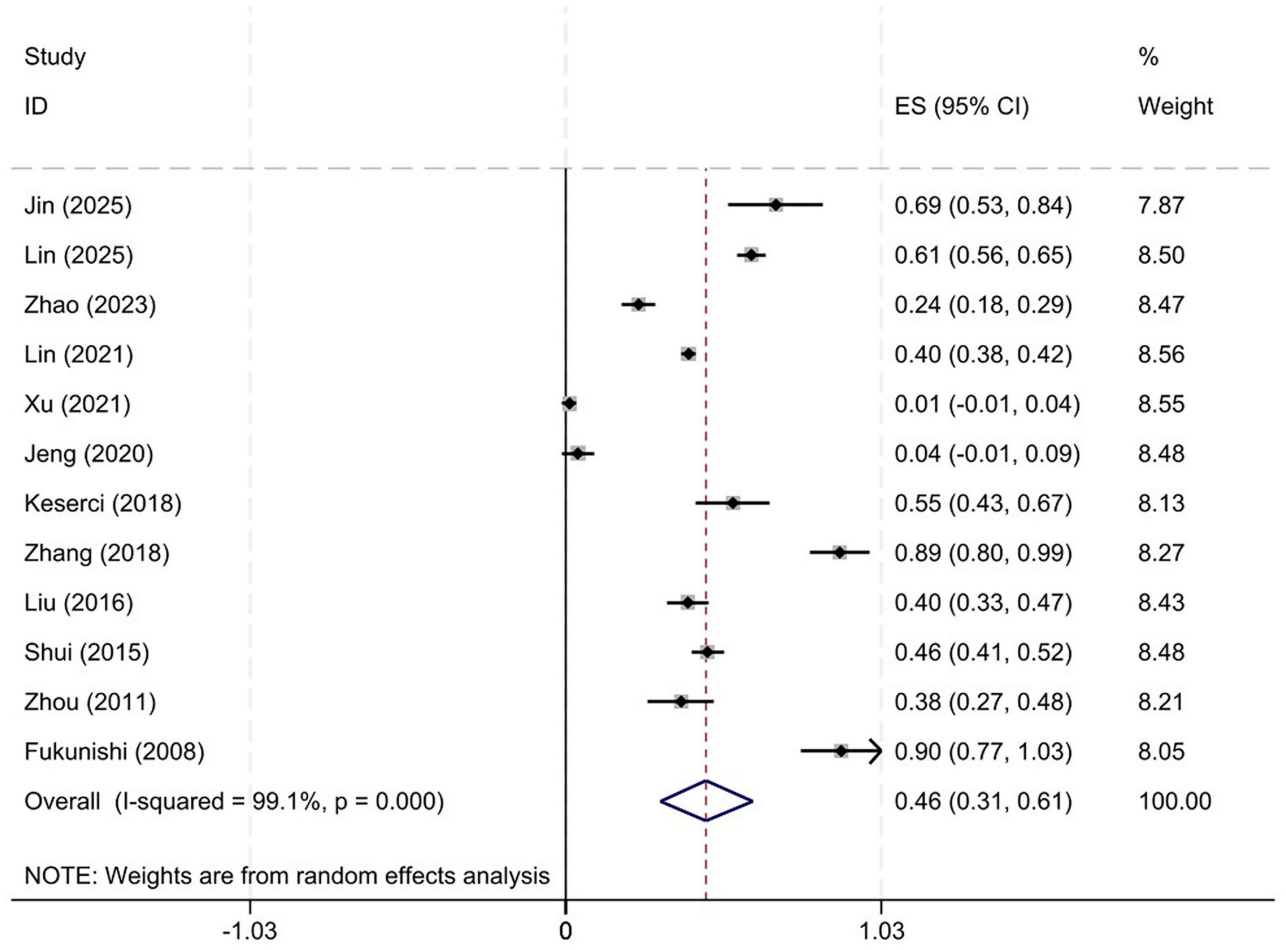
Forest plot of minor adverse events.

Twelve studies presented minor adverse events. Considering the high heterogeneity (*I*^2^ = 99.1%, *p* < 0.001), a random-effects model was performed. The overall incidence of minor adverse events was 0.46, with a 95% CI of 0.31 to 0.61 (*p* < 0.001) (Figure 6).

#### 3.5.2 Major adverse events

**Figure 7:**
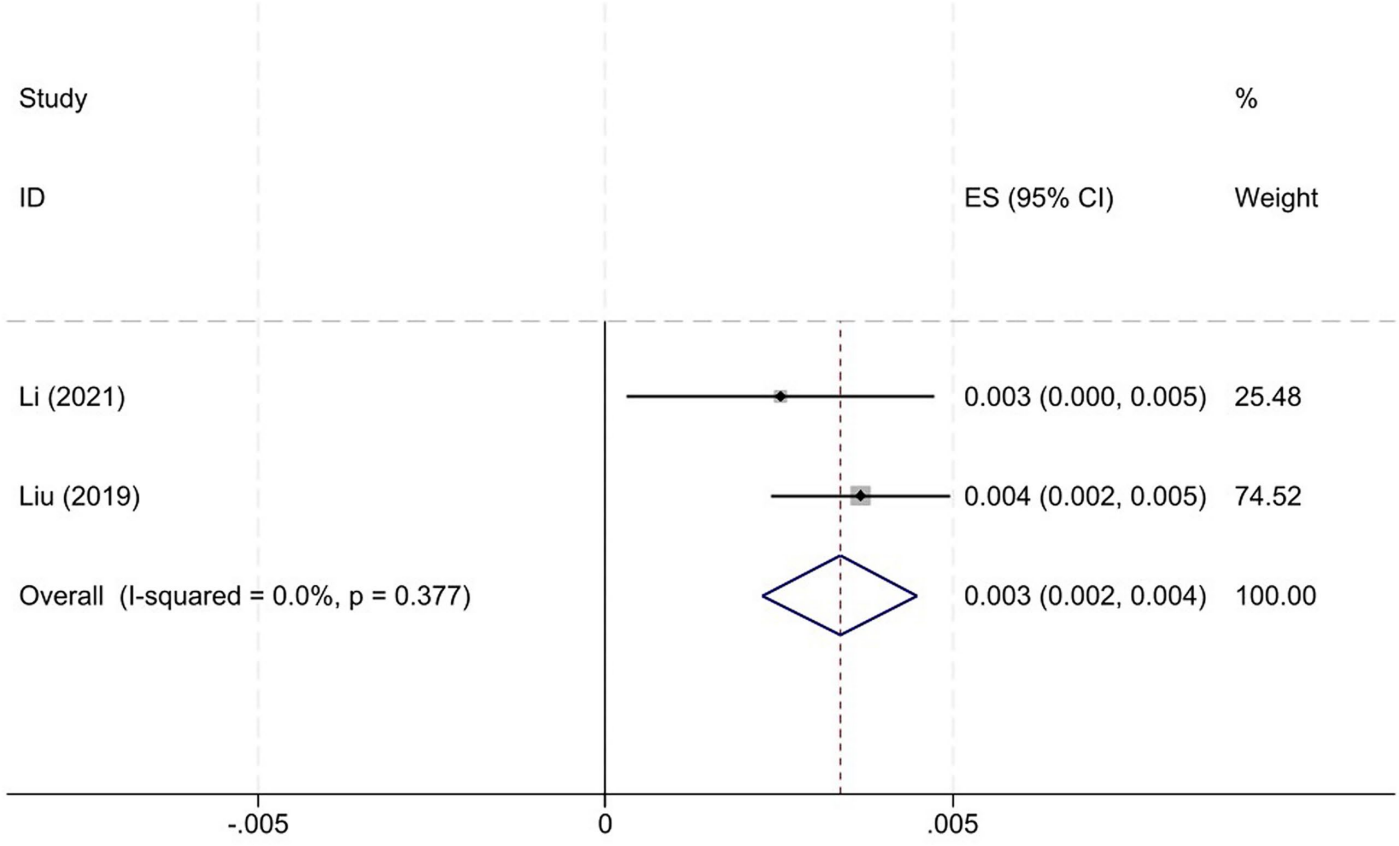
Forest plot of major adverse events.

Two studies reported major adverse events. No heterogeneity was observed (*I*^2^ = 0.0%, *p =* 0.377), and a fixed-effects model showed a pooled incidence of major adverse events was 0.003 (95% CI: 0.002, 0.004; *p <* 0.001) (Figure 7).

### 3.6 Long-term outcomes

#### 3.6.1 Reintervention rate

**Figure 8:**
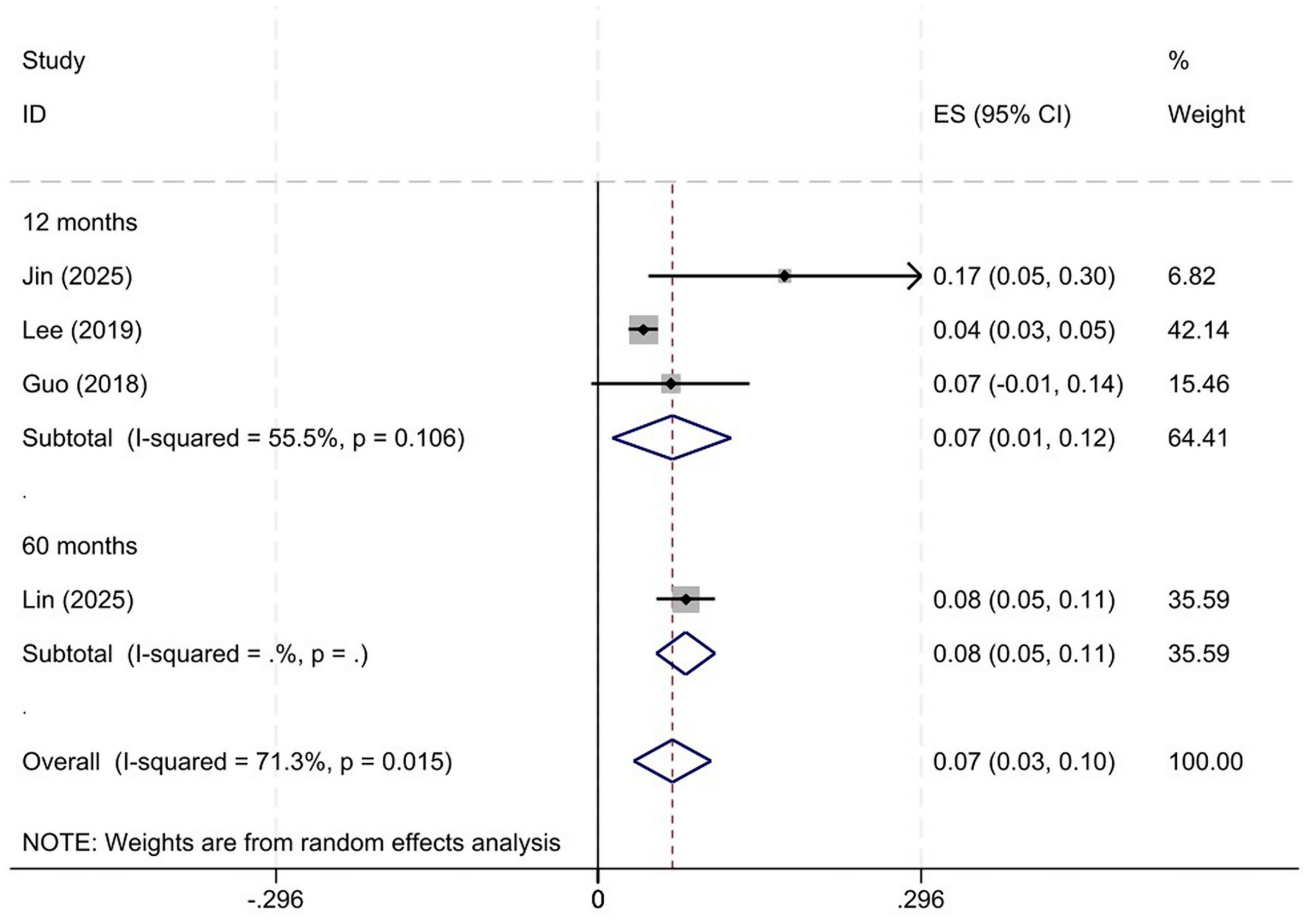
Forest plot of reintervention rate.

Four studies reported reintervention rates. At follow-up durations of 12–60 months, the reintervention rates remained relatively low. With moderate heterogeneity (*I*^2^ = 71.3%, *p =* 0.015), the random-effects model yielded a pooled rate of 0.07 (95% CI: 0.03, 0.10; *p <* 0.001). The reintervention rates at 12 and 60 months were 0.07 (95% CI: 0.01, 0.12; *p =* 0.016) and 0.08 (95% CI: 0.05, 0.11; *p <* 0.001), respectively (Figure 8).

#### 3.6.2 Recurrence rate

**Figure 9:**
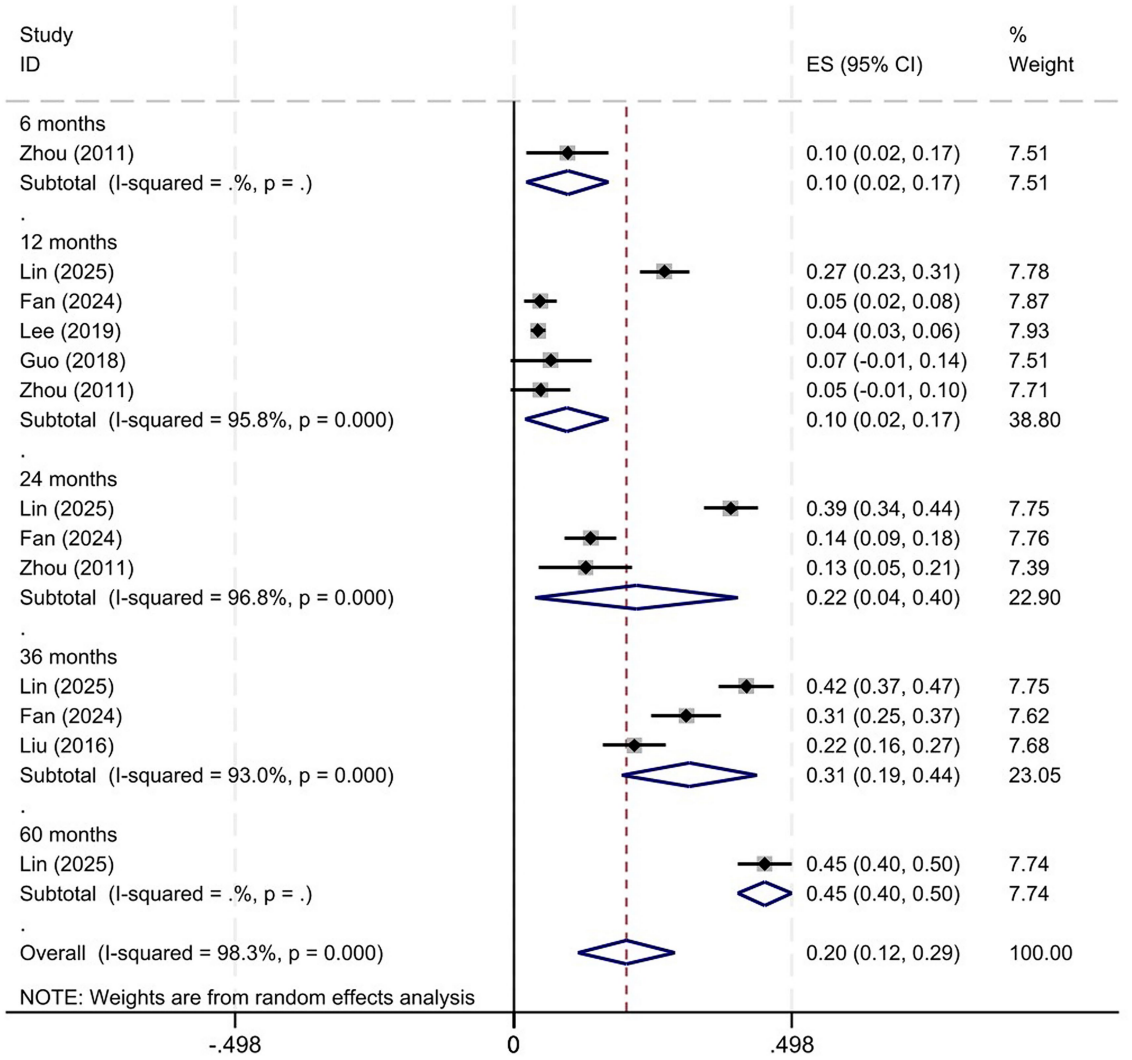
Forest plot of recurrence rate.

Seven studies reported the recurrence rates. The recurrence rate increased over time. With substantial heterogeneity (*I*^2^ = 98.3%, *p <* 0.001), a random-effects model showed a pooled recurrence rate of 0.20 (95% CI: 0.12, 0.29; *p <* 0.001). Rates at 6, 12, 24, 36, and 60 months were 0.10 (95% CI: 0.02, 0.17; *p =* 0.010), 0.10 (95% CI: 0.02, 0.17; *p =* 0.011), 0.22 (95% CI: 0.04, 0.40; *p =* 0.017), 0.31 (95% CI: 0.19, 0.44; *p <* 0.001), and 0.45 (95% CI: 0.40, 0.50; *p <* 0.001), respectively (Figure 9).

### 3.7 Pregnancy

**Figure 10:**
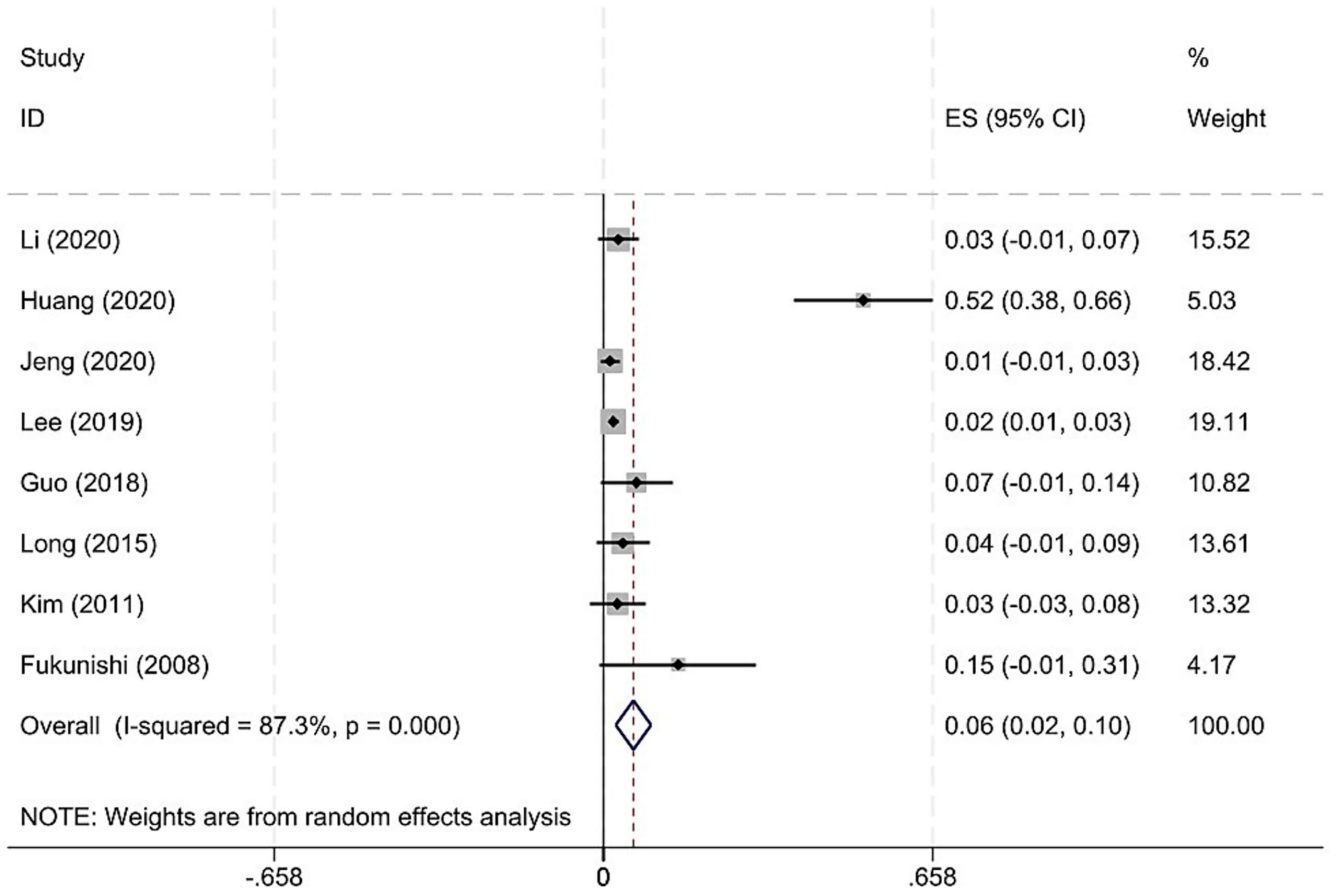
Forest plot of pregnancy rate.

Eight studies reported pregnancy outcomes. Due to substantial heterogeneity (*I*^2^ = 87.3%, *p <* 0.001), a random-effects model was used. The pooled pregnancy rate after FUAS treatment for adenomyosis was 0.06 (95% CI: 0.02, 0.10; *p <* 0.001) (Figure 10). Across the included studies, a total of 57 pregnancies were reported. Huang et al. (23) described 26 successful pregnancies after HIFU treatment, including 6 vaginal deliveries and 12 cesarean sections. Pregnancy losses included 3 missed abortions, 3 spontaneous abortions, and 2 induced abortions. Pregnancy- or delivery-related complications were reported in 5 cases, accounting for 10%. Guo et al. (24) reported 3 pregnancies, among which 1 resulted in the delivery of a healthy full-term infant. Kim et al. (25) reported 1 case of spontaneous conception followed by full-term delivery. Lee et al. (26) reported 18 pregnancies, including 5 normal spontaneous deliveries, 8 cesarean sections, and 1 preterm birth; however, neonatal outcomes were not reported separately.

All pooled results of primary and secondary outcome measures were summarized in Supplementary Tables S3, S4.

### 3.8 Sensitivity analysis

Sensitivity analysis was conducted on the primary outcomes by sequentially omitting each study to evaluate the stability of the pooled results. The overall effect sizes for dysmenorrhea VAS score, dysmenorrhea remission rate, menorrhagia severity score, menorrhagia remission rate, minor and major adverse events, and reintervention and recurrence rate remained consistent after excluding any single study, and all 95% confidence intervals were consistently positioned away from the null line. No individual study was found to exert excessive influence on the aggregated estimates, affirming the robustness and reliability of the meta-analysis outcomes. As a result, we retained all individual studies for the analysis. The findings from the sensitivity analysis are illustrated in Supplementary Figures S5–S8.

### 3.9 Publication bias

We assessed publication bias for the primary outcomes that included more than 10 studies using funnel plots and Egger’s test. The results showed no significant publication bias for the dysmenorrhea remission rate (*p* = 0.315) or minor adverse events (*p* = 0.133), whereas a significant publication bias was detected for the dysmenorrhea VAS score (*p* = 0.001). After applying the trim-and-fill method and imputing seven potentially missing studies, the pooled results remained unchanged, further supporting the robustness and stability of our findings (Supplementary Figure S9).

## 4 Discussion

This meta-analysis systematically evaluated the long-term efficacy, and reintervention outcomes of FUAS for symptomatic adenomyosis, and the results confirmed that FUAS can greatly improve the core symptoms of adenomyosis, optimize imaging and biochemical indicators, with a low incidence of serious adverse events and a favorable long-term reintervention rate.

FUAS led to a significant reduction in dysmenorrhea VAS score (WMD = 3.52, 95% CI:3.15, 3.90) and menorrhagia severity score (WMD = 1.31, 95% CI:1.21, 1.40), and the overall remission rates of dysmenorrhea and menorrhagia reached 79 and 75% respectively, which was consistent with the significant improvement of SSS score (WMD = 25.61, 95% CI:22.86, 28.37) and QOL score (WMD = 21.39, 95% CI:20.34, 22.44). In terms of sexual function, the FSFI score was significantly improved at 6 and 12 months after treatment (WMD = 5.38, 95% CI:2.77, 8.00), although there was no statistical significance at 3 months, suggesting a delayed improvement effect of FUAS on sexual function. Safety outcomes indicated that the incidence of minor adverse events was 46%, while the incidence of major adverse events was only 0.3%, reflecting the good safety of the procedure. For long-term prognosis, the long-term reintervention rate was 8%, and the recurrence rate showed a time-dependent increase, rising from 10% at 6/12 months to 45% at 60 months after treatment.

The symptomatic improvement effect of FUAS for adenomyosis observed in this study is consistent with the results of previous studies (27–29). Subgroup analyses stratified by follow-up duration were further performed for dysmenorrhea VAS scores, menorrhagia severity scores, dysmenorrhea remission rate, and menorrhagia remission rate, generating continuous pooled estimates that comprehensively reflected both short- and long-term efficacy. The results demonstrated that all these outcomes showed significant improvement compared with baseline at all follow-up time points (3–36 months), and the durability of symptom relief appeared to exceed that of some conservative medical treatments, which are often associated with a high risk of recurrence (30). The short- and long-term symptom relief rates achieved by FUAS in adenomyosis seem to be comparable to those of adenomyomectomy (31, 32). However, our findings also indicate a declining trend in symptom improvement and remission rates over time, with the dysmenorrhea and menorrhagia remission rates decreasing to 52 and 47%, respectively, at 36 months. This decline may be attributed to the progressive nature of adenomyosis and potential regrowth or persistence of incompletely ablated lesions.

For the improvement of QoL and sexual function, the results of this study showed that the QoL score was significantly improved at 3 months after treatment, while the FSFI score was improved at 6 and 12 months, which indicates that the recovery of sexual function is related to the gradual relief of chronic inflammation and the recovery of uterine volume after FUAS, and has a certain time lag (33, 34).

Imaging evaluation showed that the pooled NPVR of FUAS was 70%, and the uterine and lesion volumes were significantly reduced 3–12 months after treatment, accompanied by a significant decrease in serum CA125 level (WMD = 26.40, 95% CI:13.53, 39.28). Higher NPVR indicates better clinical efficacy with the ablation of a large volume of lesions. The marked decrease in the size of the uterus and lesions aligns with findings from a prospective study by Liu et al. (9), which demonstrated that FUAS is capable of precisely targeting and necrotizing adenomyosis lesions, thereby alleviating the uterine pressure on adjacent tissues. The significant decrease in serum CA125 levels after treatment indicates that FUAS can effectively reduce the inflammatory response of the pelvic cavity caused by adenomyosis.

HIFU treatment efficacy may be affected by symptom severity, adenomyosis classification and other factors (35–38). Zhao et al. (35) reported that type III adenomyosis achieved the highest NPVR, 74.7 ± 20.3%, compared with type I, 62.1 ± 24.1%, type II, 60.9 ± 22.0%, and type IV, 52.4 ± 19.0%, with better 6-month symptom relief. Gong et al. (36) reported that dysmenorrhea effective rates ranged from 54.7 to 100% across internal, external, full-thickness, and intramural subtypes. Xu et al. (37) found similar dysmenorrhea relief between internal and external adenomyosis (79.5% vs. 80.8%), but higher menorrhagia relief in internal disease (86.2% vs. 77.1). Collectively, focal, intramural, or well-demarcated lesions appear more amenable to complete ablation, whereas diffuse, full-thickness, or extensive external disease may have a higher risk of incomplete ablation or less durable symptom control. However, the majority of included primary studies did not systematically report clinical classification data, and the classification systems used in the small number of studies were highly different; therefore, a formal pooled subgroup analysis could not be performed. This is an important evidence gap and it should call for standardized reporting of adenomyosis classification in future FUAS studies using established criteria (39, 40).

The rate of minor adverse events observed in this study was 46%, which is somewhat higher than the 39.0% reported by Liu et al. (9). This difference might be attributed to the larger population and the inclusion of more studies that adhere to stringent adverse event reporting standards. The main minor adverse events of FUAS include transient lower abdominal pain and vaginal discharge, which are all self-limiting or can be relieved by minimal treatment (41). The safety results of this study showed that FUAS for adenomyosis has a low risk of major adverse events, which is consistent with the conclusions of most clinical studies (42). The low incidence of major adverse events (0.3%) reflects the noninvasive advantages of FUAS, which is superior to traditional surgical treatments such as adenomyomectomy (43–45). Therefore, FUAS is a safe uterus-preserving treatment for most patients with uterine adenomyosis.

The long-term risks of reintervention and recurrence following FUAS treatment for adenomyosis have attracted considerable attention. The present meta-analysis demonstrated that the overall reintervention rate was relatively low (approximately 7%), which was consistent with the findings summarized by Bahutair et al. (46) (a reintervention rate of approximately 8.2% for thermal ablation therapy) and reflects the clinical value of FUAS as a minimally invasive and noninvasive treatment for adenomyosis, especially for patients who refuse or cannot tolerate surgical treatment (47). Currently, the included studies provide limited data on the factors associated with reintervention after HIFU for adenomyosis, particularly the relationship between reintervention and adenomyosis subtype. In contrast, studies on reintervention after HIFU ablation for uterine fibroids have identified several key risk factors for subsequent treatment (48, 49). This remains an important evidence gap in the field of HIFU treatment for adenomyosis and warrants systematic investigation. Clarifying these factors would be highly relevant for clinical decision-making and patient selection.

The time-dependent increase in the recurrence rate after FUAS for adenomyosis rises from 10% at 6 months postoperatively to 45% at 60 months. This trend indicates that although FUAS can achieve favorable improvements in symptoms and quality of life, adenomyosis may still recur with extended follow-up duration. The primary explanation for this increasing recurrence over time lies in the intrinsic heterogeneous nature of adenomyotic lesions. These lesions often diffusely infiltrate the myometrium, which complicates the ability to completely target and ablate all affected tissue during FUAS treatment. Consequently, residual adenomyotic lesions may remain, capable of regrowth and subsequent recurrence of symptoms (50). Furthermore, hormonal factors play a significant role. The uterine tissue’s regenerative capacity combined with hormonal influences can contribute to the persistence or reactivation of adenomyotic lesions after ablation. This biological environment supports lesion viability and regrowth, thereby fostering recurrence even after initial successful ablation (51, 52). The recurrence rate observed in our study during 36–60 months after treatment was higher than that reported in the literature, which may be attributed to the longer follow-up duration and differences in the enrolled patient populations. Among the included studies, Fan et al. (53) reported recurrence rates of 47.6 and 30.8% for diffuse and focal adenomyosis, respectively, with a statistically significant difference. However, data on the association between adenomyosis classification and recurrence is scare. In addition, the classification criteria used across studies were inconsistent. Further high-quality studies are therefore needed to better explore the relevant influencing factors and to identify patients at higher risk of recurrence.

A key point is that recurrence is the main reason for reintervention. Recurrence may include either radiological or symptomatic recurrence. In the present study, the recurrence rate was markedly higher than the reintervention rate. This suggests that many patients with recurrence may have only mild symptoms or radiological recurrence. However, the available literature does not provide sufficient data on symptom severity among patients with recurrence. In such cases, symptoms may be controlled with conservative treatments, such as analgesics or oral contraceptives, without the need for further invasive intervention. Reintervention refers to the need for additional clinical treatment, such as repeat HIFU, hysterectomy, or LNG-IUS placement. These treatments are usually considered only when symptoms substantially impair quality of life. Previous studies have reported overall reintervention rates of approximately 4–8% after HIFU for adenomyosis (9, 54), which is broadly consistent with the reintervention rate observed in the present study. Conservative treatment for adenomyosis is unlikely to completely eliminate lesions; therefore, recurrence remains an important risk. Nevertheless, recurrence may be reduced through appropriate patient selection and adjunctive therapies.

As a uterus-sparing, minimally invasive, and noninvasive procedure, the benefits of FUAS for fertility have always been an important clinical concern. The pregnancy outcomes summarized in the present review suggest that FUAS may represent a potentially fertility-preserving option for selected patients with adenomyosis, although the evidence should be interpreted cautiously. Among the available studies, the most direct reproductive evidence comes from Huang et al. (23), who reported a pregnancy rate of 52.0% and a live birth/delivery rate of 36.0% in infertile patients with adenomyosis treated with FUAS. The pregnancy rate of 6% after FUAS in this study appears lower than that reported in several previous studies (55, 56). Most of the studies included in our analysis did not consider fertility preservation as the primary endpoint, and not all enrolled patients were willing to conceive. A systematic review by Chen et al. (57), which focused specifically on patients attempting pregnancy, reported a pregnancy rate of up to 53.4% and a live birth rate of 35.2% after FUAS. Some studies have confirmed that FUAS exerts no significant adverse effects on ovarian reserve or uterine receptivity (58), indicating that FUAS has potential value in assisting conception. Mechanistically, the potential fertility benefit of FUAS may be related to targeted thermal ablation of adenomyotic lesions while preserving the uterus, reduction of lesion and uterine volume, and partial restoration of uterine anatomy. These changes may help improve the uterine environment by reducing adenomyosis-associated inflammatory activity, improving endometrial receptivity, and normalizing abnormal uterine contractility (59, 60). A systematic evaluation revealed that pregnancy, miscarriage, and live birth rates after excisional treatment for women attempting conception were comparable to those after non-excisional treatment (HIFU, UAE, microwave ablation, and radiofrequency ablation) (61). However, Most current relevant studies are retrospective or lack a controlled design, with low-quality clinical evidence. Therefore, when counseling patients, FUAS should be presented as a uterus-sparing treatment with promising but still low-certainty reproductive evidence.

The sensitivity analyses indicated that the pooled results for the main efficacy and safety outcomes were generally stable, with no individual study exerting excessive influence on the overall estimates. Although publication bias was identified for the dysmenorrhea VAS score, the trim-and-fill analysis did not materially alter the pooled effect. However, residual heterogeneity remained for some outcomes, which may be explained by differences in FUAS device parameters, follow-up duration, and adenomyosis subtype distribution among centers. These sources of heterogeneity represent inherent limitations of meta-analyses in this clinical domain and should be taken into account when interpreting the results. Thus, while FUAS appears to be associated with meaningful symptom improvement and acceptable safety, further standardized prospective studies are warranted to strengthen the evidence base.

Overall, HIFU appears to be a safe and effective treatment option for adenomyosis, with the advantages of uterine preservation and an acceptable long-term reintervention rate. However, when treatment is considered at the individual patient level, baseline symptom severity, adenomyosis classification, and other factors may help predict personal responses to FUAS. Previous studies have preliminarily suggested that diffuse, full-thickness, or extensive external adenomyosis may be associated with a higher risk of incomplete ablation, less durable symptom control, or a higher recurrence rate. In addition, patient age, fertility desire, MRI characteristics, and other clinical factors may also contribute to the final treatment strategy. Younger patients with more active endocrine function may face a higher risk of recurrence; therefore, treatment planning in this population should emphasize both symptom control and the achievement of a high NPVR. For patients with a clear desire for future fertility, preservation of the integrity of the uterine structure should be prioritized. Therefore, clinicians should select appropriate candidates according to the type and severity of adenomyotic lesions, fertility desire, baseline symptoms, imaging features, and other factors. Standardizing FUAS operational protocols, improving ablation efficiency, integrating interventional treatment with medical therapy when appropriate, and developing individualized postoperative follow-up and management strategies may help achieve better long-term outcomes. On the other hand, evidence regarding multiple outcomes and their associated risk factors after HIFU ablation for adenomyosis remains limited. Further studies are urgently needed to identify key predictors of treatment response, recurrence, fertility outcomes, and reintervention. Establishing risk stratification models or risk scores may further support clinical decision-making and guide individualized treatment in the future.

A comparison between FUAS and classical uterus-sparing treatment, particularly adenomyomectomy, is an important issue in current adenomyosis research. Adenomyomectomy is performed laparoscopically or by laparotomy, the adenomyotic lesion is dissected or cytoreduced, and the myometrium is reconstructed with sutures (31, 62). While FUAS uses externally delivered, image-guided high-intensity focused ultrasound to generate focal thermal coagulative necrosis within adenomyotic tissue, without abdominal entry or uterine incision (63). These technical distinctions translate into different clinical strengths and limitations. The main advantage of FUAS is its noninvasive nature. It may be particularly useful in selected diffuse adenomyosis, where complete adenomyomectomy is technically difficult and carries a risk of excessive myometrial loss (62, 63). However, FUAS does not provide tissue for histological confirmation, may be limited by lesion accessibility and ablation completeness, and its efficacy may vary according to adenomyosis classification, lesion depth, and operator experience. Conversely, adenomyomectomy offers direct tissue removal, immediate histological confirmation, and may be advantageous for well-circumscribed focal adenomyosis, in which complete lesion excision is feasible (62). Over three-fourths of women experience symptom relief after conservative surgery (64). Nevertheless, adenomyomectomy is technically demanding and may be associated with blood loss, adhesion formation, myometrial weakening, uterine scar formation, and potential obstetric risks in subsequent pregnancies. Surgical series have suggested better reproductive outcomes in focal than in diffuse disease, and recurrence after conservative surgery remains heterogeneous, ranging from none to nearly 50% across studies and reaching 39.1% in a recent retrospective cohort (64–66). FUAS and adenomyomectomy should not be viewed as competing universal alternatives but as complementary uterus-sparing strategies for different clinical scenarios. Importantly, both FUAS and adenomyomectomy share a key evidence gap, as high-quality comparative or randomized trials directly comparing these modalities are lacking. A prospective randomized trial comparing FUAS with adenomyomectomy for adenomyosis, or a rigorously designed comparative cohort using propensity score matching, are needed to clarify their relative efficacy, safety, recurrence risk, reproductive outcomes, and optimal patient selection.

This study presents multiple strengths. Foremost, it is an in-depth meta-analysis that investigates the prolonged effects of FUAS on adenomyosis. It features an extended follow-up duration and offers a thorough evaluation of symptoms, sexual function, imaging, safety, long-term reintervention, and pregnancy outcomes, thereby supplying more comprehensive evidence for clinical decision-making. Second, this study featured a wide follow-up span and incorporated ultra-long-term data of 36 to 60 months, filling the gap in evidence-based medical data regarding long-term therapeutic efficacy. Third, the study analyzed the outcome indicators at different follow-up time points, which revealed the dynamic change law of the efficacy and prognosis of FUAS and has important clinical guiding significance. From a clinical perspective, FUAS may represent a valuable uterus-sparing and non-invasive treatment option for selected patients with adenomyosis, particularly those who wish to preserve the uterus, avoid abdominal surgery, or are unsuitable for conventional surgical treatment. However, HIFU potential advantages should be interpreted in the context of patient selection, disease phenotype, lesion MRI features and other risk factors.

However, this study has some limitations. First, most of the included studies were single-arm observational studies, primarily retrospective cohort studies, and randomized controlled trials with appropriate control groups were lacking. This study design may introduce potential selection bias and limit the overall level of evidence supporting the findings. Second, the number of studies reporting some outcome indicators (such as sexual function, serum CA125, and pregnancy rate) was small, which may have led to the instability of the combined results; therefore, should be interpreted with appropriate caution, and larger prospective studies with standardized reporting of reproductive and sexual function outcomes are needed. Third, the included studies were mostly from the East Asian ethnicity, and the results may have regional limitations and cannot be directly generalized to other populations; and therefore, multi-center international studies are needed. Fourth, pregnancy-related evidence should be interpreted with caution, as the studies included in the analysis of reproductive outcomes were retrospective and had small sample sizes. In addition, reporting was inconsistent across studies regarding fertility desire, spontaneous conception, miscarriage, obstetric complications, and neonatal outcomes, subgroup analysis was unable to perform. Prospective multicenter cohort studies or randomized controlled trials are therefore needed, with standardized reporting of pregnancy-related outcomes. Fifth, studies investigating multiple risk factors across different outcomes after HIFU ablation for adenomyosis remain limited. Further research is urgently needed to guide clinical practice and support individualized treatment.

Future research directions should focus on the following aspects: First, large-sample, multi-center prospective randomized controlled trials are warranted to directly compare the efficacy and safety of FUAS with other uterus-preserving treatments, including conventional medical therapy and surgical interventions. Second, MRI-based individualized therapeutic strategies should be explored. For instance, adjusting ablation parameters according to the T2 signal intensity of lesions influences efficacy and prognosis, thereby formulating an optimal surgical protocol for different subtypes of adenomyosis. Third, the combined treatment of FUAS with conservative drugs (such as GnRHa) should be explored to improve long-term efficacy and reduce recurrence rates.

## 5 Conclusion

In conclusion, FUAS serves as a noninvasive, safe, and effective treatment option for individuals with symptomatic adenomyosis, yielding long-lasting therapeutic efficacy with a favorable long-term reintervention rate. Although the recurrence rate shows a time-dependent increase, most recurrences can be reduced by patient selection, a standardized FUAS protocol, and combined treatment, and it has great potential value for patients with fertility preservation needs.

## Data Availability

The original contributions presented in the study are included in the article/Supplementary material, further inquiries can be directed to the corresponding author.

## References

[1] Zhai J Vannuccini S Petraglia F Giudice LC. Adenomyosis: mechanisms and pathogenesis. Semin Reprod Med. (2020) 38:129–43. doi: 10.1055/s-0040-1716687, 33032339 PMC7932680

[2] Wang MH Chen JH Qi XY Li ZX Huang Y. Global prevalence of adenomyosis and endometriosis: a systematic review and meta-analysis. Reprod Biol Endocrinol. (2025) 23:148. doi: 10.1186/s12958-025-01483-z, 41257733 PMC12629041

[3] Mishra I Melo P Easter C Sephton V Dhillon-Smith R Coomarasamy A. Prevalence of adenomyosis in women with subfertility: systematic review and meta-analysis. Ultrasound Obstet Gynecol. (2023) 62:23–41. doi: 10.1002/uog.26159, 36647238

[4] Kolovos G Dedes I Imboden S Mueller M. Adenomyosis-a call for awareness, early detection, and effective treatment strategies: a narrative review. Healthcare (Basel). (2024) 12:1641. doi: 10.3390/healthcare12161641, 39201199 PMC11354114

[5] Oliveira MAP Crispi CP Jr Brollo LC Crispi CP De Wilde RL. Surgery in adenomyosis. Arch Gynecol Obstet. (2018) 297:581–9. doi: 10.1007/s00404-017-4603-6, 29197987

[6] Osada H. Uterine adenomyosis and adenomyoma: the surgical approach. Fertil Steril. (2018) 109:406–17. doi: 10.1016/j.fertnstert.2018.01.032, 29566853

[7] Moawad G Youssef Y Fruscalzo A Khedhri S Faysal H Pirtea P . Effects of pretreatment strategies on fertility outcomes in patients with adenomyosis. Front Reprod Health. (2024) 6:1484202. doi: 10.3389/frph.2024.1484202, 39717429 PMC11663907

[8] Czuczwar P Stepniak A Wrona W Wozniak S Milart P Paszkowski T. The influence of uterine artery embolisation on ovarian reserve, fertility, and pregnancy outcomes - a review of literature. Prz Menopauzalny. (2016) 15:205–9. doi: 10.5114/pm.2016.65665, 28250724 PMC5327622

[9] Liu L Wang T Lei B. Image-guided thermal ablation in the management of symptomatic adenomyosis: a systematic review and meta-analysis. Int J Hyperth. (2021) 38:948–62. doi: 10.1080/02656736.2021.1939443, 34139945

[10] Moawad G Youssef Y Fruscalzo A Faysal H Kheil M Pirtea P . The present and the future of medical therapies for adenomyosis: a narrative review. J Clin Med. (2023) 12:6130. doi: 10.3390/jcm12196130, 37834773 PMC10573655

[11] Chen W Zhu H Zhang L Li K Su H Jin C . Primary bone malignancy: effective treatment with high-intensity focused ultrasound ablation. Radiology. (2010) 255:967–78. doi: 10.1148/radiol.10090374, 20501734

[12] Zhou K Strunk H Dimitrov D Vidal-Jove J Gonzalez-Carmona MA Essler M . US-guided high-intensity focused ultrasound in pancreatic cancer treatment: a consensus initiative between Chinese and European HIFU centers. Int J Hyperth. (2024) 41:2295812. doi: 10.1080/02656736.2023.2295812, 38159562

[13] Chen L Wang K Chen Z Meng Z Chen H Gao H . High intensity focused ultrasound ablation for patients with inoperable liver cancer. Hepato-Gastroenterology. (2015) 62:140–3. 25911884

[14] Kang Y Sang C Zhao L Ding K Zhao S. High-intensity focused ultrasound combined with hysteroscopic insertion of levonorgestrel-releasing intrauterine system for intrinsic adenomyosis: a retrospective observational study. Int J Hyperth. (2025) 42:2531028. doi: 10.1080/02656736.2025.2531028, 40641223

[15] Liu R Hao H Yin Y Chen L Liu Y. Effect of high-intensity focused ultrasound on different types of Adenomyosis based on magnetic resonance imaging classification. J Ultrasound Med. (2024) 43:1947–55. doi: 10.1002/jum.16529, 39032000

[16] Lee KW Lee CL. An alternative treatment for uterine fibroids and adenomyosis: high-intensity focused ultrasound. Gynecol Minim Invasive Ther. (2023) 12:61–3. doi: 10.4103/gmit.gmit_20_23, 37416107 PMC10321341

[17] Page MJ McKenzie JE Bossuyt PM Boutron I Hoffmann TC Mulrow CD . The PRISMA 2020 statement: an updated guideline for reporting systematic reviews. BMJ. (2021) 372:n71. doi: 10.1136/bmj.n71 33782057 PMC8005924

[18] Sacks D McClenny TE Cardella JF Lewis CA. Society of interventional radiology clinical practice guidelines. J Vasc Interv Radiol. (2003) 14:S199–202. doi: 10.1097/01.rvi.0000094584.83406.3e, 14514818

[19] Harding G Coyne KS Thompson CL Spies JB. The responsiveness of the uterine fibroid symptom and health-related quality of life questionnaire (UFS-QOL). Health Qual Life Outcomes. (2008) 6:99. doi: 10.1186/1477-7525-6-99, 19014505 PMC2603004

[20] Slim K Nini E Forestier D Kwiatkowski F Panis Y Chipponi J. Methodological index for non-randomized studies (minors): development and validation of a new instrument. ANZ J Surg. (2003) 73:712–6. doi: 10.1046/j.1445-2197.2003.02748.x, 12956787

[21] Miller CE Osman KM. Transcervical radiofrequency ablation of symptomatic uterine fibroids: 2-year results of the SONATA pivotal trial. J Gynecol Surg. (2019) 35:345–9. doi: 10.1089/gyn.2019.0012, 32226268 PMC7099422

[22] Shui L Mao S Wu Q Huang G Wang J Zhang R . High-intensity focused ultrasound (HIFU) for adenomyosis: two-year follow-up results. Ultrason Sonochem. (2015) 27:677–81. doi: 10.1016/j.ultsonch.2015.05.024, 26050604

[23] Huang YF Deng J Wei XL Sun X Xue M Zhu XG . A comparison of reproductive outcomes of patients with adenomyosis and infertility treated with high-intensity focused ultrasound and laparoscopic excision. Int J Hyperth. (2020) 37:301–7. doi: 10.1080/02656736.2020.1742390, 32208771

[24] Guo Q Xu F Ding Z Li P Wang X Gao B. High intensity focused ultrasound treatment of adenomyosis: a comparative study. Int J Hyperth. (2018) 35:505–9. doi: 10.1080/02656736.2018.1509238, 30306813

[25] Kim KA Yoon SW Lee C Seong SJ Yoon BS Park H. Short-term results of magnetic resonance imaging-guided focused ultrasound surgery for patients with adenomyosis: symptomatic relief and pain reduction. Fertil Steril. (2011) 95:1152–5. doi: 10.1016/j.fertnstert.2010.09.024, 20970127

[26] Lee JS Hong GY Lee KH Song JH Kim TE. Safety and efficacy of ultrasound-guided high-intensity focused ultrasound treatment for uterine fibroids and Adenomyosis. Ultrasound Med Biol. (2019) 45:3214–21. doi: 10.1016/j.ultrasmedbio.2019.08.022, 31563479

[27] Tang Y Hu WH Wang H Wu J Wen MB Su B . Magnetic resonance imaging-based classification Systems for Informing Better Outcomes of Adenomyosis after ultrasound-guided high-intensity focused ultrasound ablating surgery. J Magn Reson Imaging. (2024) 59:1787–97. doi: 10.1002/jmri.28943, 37671487

[28] Yao R Zhao W Gao B Hu J Wang T. Microbubble contrast agent SonoVue combined with oxytocin improves the efficiency of high-intensity focused ultrasound ablation for adenomyosis. Int J Hyperth. (2021) 38:1601–8. doi: 10.1080/02656736.2021.1993357, 34763594

[29] Cheung VY. Current status of high-intensity focused ultrasound for the management of uterine adenomyosis. Ultrasonography. (2017) 36:95–102. doi: 10.14366/usg.16040, 28145109 PMC5381845

[30] Vannuccini S Luisi S Tosti C Sorbi F Petraglia F. Role of medical therapy in the management of uterine adenomyosis. Fertil Steril. (2018) 109:398–405. doi: 10.1016/j.fertnstert.2018.01.013, 29566852

[31] Zhu L Chen S Che X Xu P Huang X Zhang X. Comparisons of the efficacy and recurrence of adenomyomectomy for severe uterine diffuse adenomyosis via laparotomy versus laparoscopy: a long-term result in a single institution. J Pain Res. (2019) 12:1917–24. doi: 10.2147/JPR.S205561, 31303783 PMC6603287

[32] Sun AJ Luo M Wang W Chen R Lang JH. Characteristics and efficacy of modified adenomyomectomy in the treatment of uterine adenomyoma. Chin Med J. (2011) 124:1322–6. 21740741

[33] Evangelista A Dantas T Zendron C Soares T Vaz G Oliveira MA. Sexual function in patients with deep infiltrating endometriosis. J Sex Med. (2014) 11:140–5. doi: 10.1111/jsm.12349, 24165172

[34] Wang X Qin J Wang L Chen J Chen W Tang L. Effect of high-intensity focused ultrasound on sexual function in the treatment of uterine fibroids: comparison to conventional myomectomy. Arch Gynecol Obstet. (2013) 288:851–8. doi: 10.1007/s00404-013-2775-2, 23564052

[35] Zhao Y Luo S Liu Y He Y Liu X Guohua H . High intensity focused ultrasound treatment for adenomyosis: comparison of efficacy based on MRI features. Int J Hyperth. (2023) 40:2197574. doi: 10.1080/02656736.2023.2197574, 37031960

[36] Gong C Wang Y Lv F Zhang L Wang Z. Evaluation of high intensity focused ultrasound treatment for different types of adenomyosis based on magnetic resonance imaging classification. Int J Hyperth. (2022) 39:530–8. doi: 10.1080/02656736.2022.2052366, 35300545

[37] Xu F Lin Z Wang Y Gong C He M Guo Q . Comparison of high-intensity focused ultrasound for the treatment of internal and external adenomyosis based on magnetic resonance imaging classification. Int J Hyperth. (2023) 40:2211268. doi: 10.1080/02656736.2023.2211268, 37202156

[38] Keserci B Duc NM. Magnetic resonance imaging features influencing high-intensity focused ultrasound ablation of adenomyosis with a nonperfused volume ratio of ≥90% as a measure of clinical treatment success: retrospective multivariate analysis. Int J Hyperth. (2018) 35:626–36. doi: 10.1080/02656736.2018.1516301, 30307340

[39] Kishi Y Suginami H Kuramori R Yabuta M Suginami R Taniguchi F. Four subtypes of adenomyosis assessed by magnetic resonance imaging and their specification. Am J Obstet Gynecol. (2012) 207:114.e1–7. doi: 10.1016/j.ajog.2012.06.027, 22840719

[40] Gordts S Grimbizis G Campo R. Symptoms and classification of uterine adenomyosis, including the place of hysteroscopy in diagnosis. Fertil Steril. (2018) 109:380–8.e1. doi: 10.1016/j.fertnstert.2018.01.006, 29566850

[41] Liu Y Zhang WW He M Gong C Xie B Wen X . Adverse effect analysis of high-intensity focused ultrasound in the treatment of benign uterine diseases. Int J Hyperth. (2018) 35:56–61. doi: 10.1080/02656736.2018.1473894, 29792359

[42] Chen J Chen W Zhang L Li K Peng S He M . Safety of ultrasound-guided ultrasound ablation for uterine fibroids and adenomyosis: a review of 9988 cases. Ultrason Sonochem. (2015) 27:671–6. doi: 10.1016/j.ultsonch.2015.05.031, 26093678

[43] Ioannidou A Louis K Sioutis D Panagopoulos P Theofanakis C Machairiotis N. Conservative surgical management of adenomyosis: implications for infertility and pregnancy outcomes-a perspective review. J Clin Med. (2025) 14:6956. doi: 10.3390/jcm14196956, 41096036 PMC12525121

[44] Athanasiou A Fruscalzo A Dedes I Mueller MD Londero AP Marti C . Advances in adenomyosis treatment: high-intensity focused ultrasound, percutaneous microwave therapy, and radiofrequency ablation. J Clin Med. (2024) 13:5828. doi: 10.3390/jcm13195828, 39407887 PMC11476787

[45] Ahn JW You SG Go EB Lee SH Kim JS Cho HJ . Minimally invasive adenomyomectomy via a laparoscopic-assisted approach compared to a laparoscopic or laparotomic approach. Taiwan J Obstet Gynecol. (2021) 60:1005–10. doi: 10.1016/j.tjog.2021.09.010, 34794729

[46] Bahutair SN Alhubaishi LY. High-intensity focused ultrasound in adenomyosis treatment: insights on safety, efficacy, and reproductive prospects. Womens Health (Lond). (2024) 20:17455057241295593. doi: 10.1177/17455057241295593, 39494764 PMC11536486

[47] Chen J Porter AE Kho KA. Current and future surgical and interventional management options for Adenomyosis. Semin Reprod Med. (2020) 38:157–67. doi: 10.1055/s-0040-1718921, 33152768

[48] Inbar Y Rabinovici J Sverdlove R Ziv-Baran T Machtinger R. Long-term outcomes and re-intervention rates in women undergoing MRI-guided focused ultrasound (MRgFUS) for uterine fibroids: a 7-year follow-up study. J Assist Reprod Genet. (2025) 42:1191–6. doi: 10.1007/s10815-025-03405-9, 39899259 PMC12055703

[49] Dou Y Zhang L Liu Y He M Wang Y Wang Z. Long-term outcome and risk factors of reintervention after high intensity focused ultrasound ablation for uterine fibroids: a systematic review and meta-analysis. Int J Hyperth. (2024) 41:2299479. doi: 10.1080/02656736.2023.2299479, 38164630

[50] Pang LL Mei J Fan LX Zhao TT Li RN Wen Y. Efficacy of high-intensity focused ultrasound combined with GnRH-a for adenomyosis: a systematic review and meta-analysis. Front Public Health. (2021) 9:688264. doi: 10.3389/fpubh.2021.688264, 34485218 PMC8415267

[51] Capezzuoli T Toscano F Ceccaroni M Roviglione G Stepniewska A Fambrini M . Conservative surgical treatment for adenomyosis: new options for looking beyond uterus removal. Best Pract Res Clin Obstet Gynaecol. (2024) 95:102507. doi: 10.1016/j.bpobgyn.2024.102507, 38906739

[52] Guo SW. Cracking the enigma of adenomyosis: an update on its pathogenesis and pathophysiology. Reproduction. (2022) 164:R101–21. doi: 10.1530/REP-22-0224, 36099328

[53] Fan LX Zhang Y Yang LL Ji XL Wang Y Huang YF . Analysis of related factors influencing postoperative recurrence of adenomyosis treated with HIFU. Arch Gynecol Obstet. (2024) 309:1765–73. doi: 10.1007/s00404-023-07340-x, 38347252

[54] Liu L Tian H Lin D Zhao L Wang H Hao Y. Risk of recurrence and reintervention after uterine-sparing interventions for symptomatic Adenomyosis: a systematic review and Meta-analysis. Obstet Gynecol. (2023) 141:711–23. doi: 10.1097/AOG.0000000000005080, 36897132 PMC10026977

[55] Wei J Wang L Tao H Wang X Zheng F He P . Comparison of pregnancy outcomes in infertile patients with different types of adenomyosis treated with high-intensity focused ultrasound. Int J Hyperth. (2023) 40:2238140. doi: 10.1080/02656736.2023.2238140, 37495217

[56] Haiyan S Lin W Shuhua H Wang W. High-intensity focused ultrasound (HIFU) combined with gonadotropin-releasing hormone analogs (GnRHa) and levonorgestrel-releasing intrauterine system (LNG-IUS) for adenomyosis: a case series with long-term follow up. Int J Hyperth. (2019) 36:1178–84. doi: 10.1080/02656736.2019.1679892, 31793356

[57] Chen Y Lin S Xie X Yi J Liu X Guo SW. Systematic review and meta-analysis of reproductive outcomes after high-intensity focused ultrasound (HIFU) treatment of adenomyosis. Best Pract Res Clin Obstet Gynaecol. (2024) 92:102433. doi: 10.1016/j.bpobgyn.2023.102433, 38065008

[58] Lee JS Hong GY Lee KH Kim TE. Changes in anti-müllerian hormone levels as a biomarker for ovarian reserve after ultrasound-guided high-intensity focused ultrasound treatment of adenomyosis and uterine fibroid. BJOG. (2017) 124:18–22. doi: 10.1111/1471-0528.14739, 28856867

[59] Zhang G Li L Sun M Yu X. Progress in high intensity focused ultrasound ablation for fertility preservation therapy of uterine fibroids and Adenomyosis. Reprod Sci. (2025) 32:15–25. doi: 10.1007/s43032-024-01745-y, 39532767 PMC11729086

[60] Wu HM Tsai TC Liu SM Pai AH Chen LH. The current understanding of molecular mechanisms in adenomyosis-associated infertility and the treatment strategy for assisted reproductive technology. Int J Mol Sci. (2024) 25:8937. doi: 10.3390/ijms25168937, 39201621 PMC11354813

[61] Jiang L Han Y Song Z Li Y. Pregnancy outcomes after uterus-sparing operative treatment for adenomyosis: a systematic review and Meta-analysis. J Minim Invasive Gynecol. (2023) 30:543–54. doi: 10.1016/j.jmig.2023.03.015, 36972750

[62] Grimbizis GF Mikos T Tarlatzis B. Uterus-sparing operative treatment for adenomyosis. Fertil Steril. (2014) 101:472–487.e8. doi: 10.1016/j.fertnstert.2013.10.025, 24289992

[63] Zhang L Rao F Setzen R. High intensity focused ultrasound for the treatment of adenomyosis: selection criteria, efficacy, safety and fertility. Acta Obstet Gynecol Scand. (2017) 96:707–14. doi: 10.1111/aogs.13159, 28437003

[64] Younes G Tulandi T. Conservative surgery for Adenomyosis and results: a systematic review. J Minim Invasive Gynecol. (2018) 25:265–76. doi: 10.1016/j.jmig.2017.07.014, 28739414

[65] Tan J Moriarty S Taskin O Allaire C Williams C Yong P . Reproductive outcomes after fertility-sparing surgery for focal and diffuse Adenomyosis: a systematic review. J Minim Invasive Gynecol. (2018) 25:608–21. doi: 10.1016/j.jmig.2017.12.020, 29305234

[66] Lu K Zhong G Lian B Zhong X Xie M Wu Y. Recurrence rates and associated risk factors after conservative surgery for adenomyosis: a retrospective study. BMC Womens Health. (2024) 24:619. doi: 10.1186/s12905-024-03457-6, 39578802 PMC11583534

[67] Zhao X Zhang W Mi M Wang H Wang Y Feng L . Comparative study of female sexual function in adenomyosis patients who received the treatment of intensity-focused ultrasound ablation or laparoscopic total hysterectomy. Int J Gynaecol Obstet. (2026). doi: 10.1002/ijgo.70824, 41549913

[68] Lin J Yang Z Wang L Xiao Z Tan T Chen J. Efficacy of focused ultrasound ablation surgery in patients with adenomyosis and coexisting pelvic adhesions. Int J Hyperth. (2025) 42:2461456. doi: 10.1080/02656736.2025.2461456, 39947638

[69] Jin Z Wang L Wang D Zheng Q Qing X Zhang Y. Innovative application of high-intensity focused ultrasound combined with endometrial thermal balloon ablation in the treatment of adenomyosis: a cohort study. Eur J Obstet Gynecol Reprod Biol. (2025) 307:134–41. doi: 10.1016/j.ejogrb.2025.01.049, 39914106

[70] Wu S Liu J Liu X Han Y. High-intensity focused ultrasound for endometrial ablation in adenomyosis: a clinical study. Front Med (Lausanne). (2024) 11:1332080. doi: 10.3389/fmed.2024.1332080, 38576714 PMC10991773

[71] Zhu H Ma Q Dong G Yang L Li Y Song S . Clinical evaluation of high-intensity focused ultrasound ablation combined with mifepristone and levonorgestrel-releasing intrauterine system to treat symptomatic adenomyosis. Int J Hyperth. (2023) 40:2161641. doi: 10.1080/02656736.2022.2161641, 36586419

[72] Xu Y Zhou Z Wang H Shao L Liu G. High-intensity focused ultrasound combined with gonadotropin-releasing hormone agonist or levonorgestrel-releasing intrauterine system in treating dysmenorrhea of severe adenomyosis. J Comput Assist Tomogr. (2021) 45:224–31. doi: 10.1097/RCT.0000000000001138, 33661158

[73] Li X Zhu X He S Jiang Z Li H Tian X . High-intensity focused ultrasound in the management of adenomyosis: long-term results from a single center. Int J Hyperth. (2021) 38:241–7. doi: 10.1080/02656736.2021.1886347, 33602049

[74] Li W Mao J Liu Y Zhu Y Li X Zhang Z . Clinical effectiveness and potential long-term benefits of high-intensity focused ultrasound therapy for patients with adenomyosis. J Int Med Res. (2020) 48:300060520976492. doi: 10.1177/0300060520976492, 33349096 PMC7758569

[75] Jeng CJ Ou KY Long CY Chuang L Ker CR. 500 cases of high-intensity focused ultrasound (HIFU) ablated uterine fibroids and adenomyosis. Taiwan J Obstet Gynecol. (2020) 59:865–71. doi: 10.1016/j.tjog.2020.09.013, 33218403

[76] Xiao-Ying Z Ying-Shu G Jiu-Mei C Jin-Juan W Hong Y Chun-Yi Z . Effect of pre-treatment with gonadotropin-releasing hormone analogue GnRH-α on high-intensity focussed ultrasound ablation for diffuse adenomyosis: a preliminary study. Int J Hyperth. (2018) 34:1289–97. doi: 10.1080/02656736.2018.1440014, 29447020

[77] Feng Y Hu L Chen W Zhang R Wang X Chen J. Safety of ultrasound-guided high-intensity focused ultrasound ablation for diffuse adenomyosis: a retrospective cohort study. Ultrason Sonochem. (2017) 36:139–45. doi: 10.1016/j.ultsonch.2016.11.022, 28069193

[78] Feng Y Chen J Hu L Liu C Wang X Chen W. Short-term and long-term efficacy of HIFU ablation for diffuse and focal adenomyosis. Chin J Interv Imaging Ther. (2017) 14:22–6. doi: 10.13929/j.1672-8475.201608042

[79] Liu X Wang W Wang Y Wang Y Li Q Tang J. Clinical predictors of long-term success in ultrasound-guided high-intensity focused ultrasound ablation treatment for adenomyosis: a retrospective study. Medicine (Baltimore). (2016) 95:e2443. doi: 10.1097/MD.0000000000002443, 26817877 PMC4998251

[80] Long L Chen J Xiong Y Zou M Deng Y Chen L . Efficacy of high-intensity focused ultrasound ablation for adenomyosis therapy and sexual life quality. Int J Clin Exp Med. (2015) 8:11701–7. 26380007 PMC4565390

[81] Zhang X Li K Xie B He M He J Zhang L. Effective ablation therapy of adenomyosis with ultrasound-guided high-intensity focused ultrasound. Int J Gynaecol Obstet. (2014) 124:207–11. doi: 10.1016/j.ijgo.2013.08.022, 24380611

[82] Zhou M Chen JY Tang LD Chen WZ Wang ZB. Ultrasound-guided high-intensity focused ultrasound ablation for adenomyosis: the clinical experience of a single center. Fertil Steril. (2011) 95:900–5. doi: 10.1016/j.fertnstert.2010.10.020, 21067723

[83] Fukunishi H Funaki K Sawada K Yamaguchi K Maeda T Kaji Y. Early results of magnetic resonance-guided focused ultrasound surgery of adenomyosis: analysis of 20 cases. J Minim Invasive Gynecol. (2008) 15:571–9. doi: 10.1016/j.jmig.2008.06.010, 18657480

